# Plant uptake of nitrogen adsorbed to biochars made from dairy manure

**DOI:** 10.1038/s41598-021-94337-8

**Published:** 2021-07-22

**Authors:** Leilah Krounbi, Akio Enders, John Gaunt, Margaret Ball, Johannes Lehmann

**Affiliations:** 1 Soil and Crop Sciences, School of Integrative Plant Science, College of Agriculture and Life Sciences, Cornell University, Ithaca, NY 14853 USA; 2 bio365 LLC, Ithaca, NY 14850 USA; 3 Cornell Atkinson Center for Sustainability, Cornell University, Ithaca, NY 14853 USA

**Keywords:** Biogeochemistry, Element cycles

## Abstract

The conversion of dairy waste with high moisture contents to dry fertilizers may reduce environmental degradation while lowering crop production costs. We converted the solid portion of screw-pressed dairy manure into a sorbent for volatile ammonia (NH_3_) in the liquid fraction using pyrolysis and pre-treatment with carbon dioxide (CO_2_). The extractable N in manure biochar exposed to NH_3_ following CO_2_ pre-treatment reached 3.36 g N kg^−1^, 1260-fold greater extractable N than in untreated manure biochar. Ammonia exposure was 142-times more effective in increasing extractable N than immersing manure biochar in the liquid fraction containing dissolved ammonium. Radish and tomato grown in horticultural media with manure biochar treated with CO_2_ + NH_3_ promoted up to 35% greater plant growth (dry weight) and 36–83% greater N uptake compared to manure biochar alone. Uptake of N was similar between plants grown with wood biochar exposed to CO_2_ + NH_3_, compared to N-equivalent treatments. The available N in dairy waste in New York (NY) state, if pyrolyzed and treated with NH_3_ + CO_2_, is equivalent to 11,732–42,232 Mg N year^−1^, valued at 6–21.5 million USD year^−1^. Separated dairy manure treated with CO_2_ + NH_3_ can offset 23–82% of N fertilizer needs of NY State, while stabilizing both the solid and liquid fraction of manure for reduced environmental pollution.

## Introduction

Nitrogen (N) management is a major challenge in agricultural systems. Nitrogen fertilizer for crops is a significant cost, while the disposal of N-rich wastes such as dairy manure, can also be costly. The shift toward sustainable farming highlights the need for opportunistic waste management able to capture nutrients from liquid waste streams and transform them into high-value, dry fertilizers that can be safely transported and traded.

Dairy manure excretion in NY State alone averages 12,821,616 Mg per year^1,2^. Containing approximately 64,108 Mg N, 16,786 Mg P, 44,876 Mg K^3^, these excreted nutrients are sufficient to fertilize the state’s extensive 17,321 hectares (ha) of corn production, requiring 51,360 Mg N calculated using the average application rate in 2018, 96 kg ha^−1^^4–6^. The benefits incurred by transforming dairy waste into agronomic inputs extend to dairy farmers and grain farmers alike in NY State; a farmer growing 81 ha of corn spends 28,000 USD year^−1^ for fertilizer^7^, while a dairy farmer with 550 cows spends 25,000 USD year^−1^ for manure storage^8^. To couple these processes, new technologies for recycling dairy waste products back into crop nutrient inputs are necessary.

While direct manure spreading has been the most common means of disposal and re-use of dairy waste, such practices can result in transport of N and P into waterways^9,10^, as nutrients from manure often exceed the agronomic demand^11^. Furthermore, manure storage for future manure application produces notable quantities of methane, a known greenhouse gas^5,12^. Moreover, long-distance transport of dairy manure for land application also has a number of drawbacks such as the spreading of pathogens^13^ and costs associated with transporting a material with a water content of > 70%^14^. Alternatives to direct land application of dairy slurry are needed with less detrimental impact on the environment^15^, which utilize the high nutrient content of manure for agronomic purposes. Source-separation of manure followed by anaerobic digestion of the liquid portion is one alternative^16–18^ that is being adopted across farms of all scales in NY State^19^ and other regions.

Solid–liquid separation of manure is a first step in efficient re-use of waste nutrients^16^. The physical separation of manure into solid and liquid fractions (slurry) significantly lowers N leaching from the solid fraction^17,18^. Most of the inorganic N in dairy manure is found in the liquid portion, approximately 4400 mg NH_4_–N L^−1^^17^, and can be a significant source of N_2_O and NH_3_ emissions from lagoons^5,20,21^. Thus, a technology is needed for removing N from stored slurry and converting it into a fertilizer. We see great potential in converting the solid portion of separated dairy manure into a biological charcoal, or biochar, with high sorption properties, able to remove N from the liquid portion of dairy manure.

Pyrolysis is one technology which can transform residual biomass into highly porous, surface-functionalized adsorbents^22^. The temperatures used in pyrolysis (typically between 400 and 700 °C) assure full sterilization of manure, along with densification and desiccation, leading to safer, and cheaper transportation per unit dry material^14,15^. Much of the literature on biochar sorbents refer to high surface area of low ash biochars^23,24^ derived from plants^25–29^. Increasing concern for the environmental burden of manure from animal production has highlighted the relevance of manure biochars as agricultural amendments^30–33^. As pyrolysis requires feedstocks to have a moisture content below 15%, a number of strategies have been implemented for reducing the moisture content of manure, such as co-pyrolysis with woody feedstocks, or utilization of the thermochemical byproducts such as emitted heat or bio-oils for drying of moist manures^34,35^.

Manure biochars are notably rich in plant-essential nutrients such as phosphorus and potassium^23,36^, as well as total N^37–39^. They also have the ability to adsorb residual ammonium (NH_4_)^27^ and volatile ammonia gas (NH_3_)^29^ and can enhance plant N-use efficiency^40^. The ability to sorb NH_3_ increases after pre-exposure to carbon dioxide (CO_2_)^41^. Since CO_2_ is a by-product of the pyrolysis process^42^, biochars made from the solid portion of dairy manure can be treated with CO_2_ before using them to sorb volatile NH_3_ from slurry lagoons.

Ammonia gas is reported to sorb onto woody biochar and CO_2_-doped human manure biochar via both strong and weak mechanisms^27,29,40^, pointing to both short and long-term plant availability. Sorbed N from cattle urine onto woody biochar was reported as plant-available, promoting the growth of rye grass^43^. Yet no study has evaluated the actual plant-availability of NH_3_ sorbed onto manure biochar after exposure to CO_2_.

While surface charge and acid–base interactions drive N interactions with biochar made from woody feedstocks, precipitation of N salts such as ammonium bicarbonate (NH_4_HCO_3_) is a more likely mechanism for N removal by ash-rich dairy manure digestate biochar. Moreover, NH_3_ loading via NH_4_HCO_3_ precipitation may exceed monolayer surface adsorption to multi-layered sorption through intermittent exposure to CO_2_^44^. Recent work demonstrated that exposure of biochar to NH_3_ re-functionalizes surfaces with amine groups^29^ which are then able to adsorb CO_2_^41,44–46^. The incorporation of CO_2_ molecules may further enhance NH_3_ retention through the formation of NH_4_HCO_3_, creating NH_4_HCO_3_-intercalated biochar for use as a mineral-organic, slow-release fertilizer. The intimate association between the first layer of chemisorbed NH_3_ on biochar surfaces and NH_4_HCO_3_ precipitates projecting further out from the surface is expected to provide both long-term and immediately available N. Yet no study has evaluated the plant-availability of N incorporated into manure biochar through sequential NH_3_ and CO_2_ adsorption.

Therefore, we quantify the plant uptake of N adsorbed to dairy manure biochar using either liquid NH_4_^+^ or gaseous NH_3_ with prior CO_2_ conditioning. Crops grown in the greenhouse, tomato, marigold, and radish, were used in this small-scale study to demonstrate proof of concept. We benchmarked the performance of dairy manure biochar as an adsorber against wood biochar, a material reported to sorb up to 6 mg g^−1^ NH_3_-N^43,47^. We compare the plant-availability of N from biochars exposed to either liquid NH_4_^+^ or gaseous NH_3_ to the availability of N from urea fertilizer added in combination with each biochar. We expect greater plant-availability of N incorporated to dairy manure biochars compared to that incorporated into wood biochar. We expect greater N use efficiency from both biochars exposed to N compared to biochar added with inorganic fertilizer.

## Results

The increase in total N of the amendments following NH_3_ exposure was much larger for wood (1.13% point change in N, from 0.75 to 1.88% N) than manure biochars (a point change of 0.06% N, from 2.05 to 2.11% N). The increase in KCl-extractable N (sum of NO_3_^–^N and NH_4_^+^-N) was similar between wood (0.005–3.1 g N kg^−1^) and manure biochars (0.026–3.4 g N kg^−1^). The KCl-extractable, or plant-available N in manure biochar following NH_3_ exposure increased 127 fold, in comparison to a 595 fold increase for wood biochar, with only 27% of the added N in wood biochar following NH_3_ exposure being plant-available. This increase in plant-available N versus total N following NH_3_ exposure was 21-fold greater for manure than wood biochar (Table 1). Furthermore, the increase in total N in manure biochar exposed to NH_3_ (0.06%-points), was smaller than the increase in plant-available N (0.34%-points) (Table 1). Exposure to NH_3_ therefore increased the plant-available N in both biochars to the same extent, apparently, irrespective of the total N increase.

**Table 1:** Total carbon and nitrogen and KCl-extractable ammonium (NH_4_^+^-N) and nitrate (NO_3_^–^N) in amendments used for the greenhouse trial ± the standard deviation.

Analysis	Unit	Wood biochar	Manure biochar	Slurry	Wood biochar NH_3_ + CO_2_	Manure biochar CO_2_ + NH_3_	Wood biochar + slurry	Manure biochar + slurry	Potting media (peat)
Nitrogen	% (w w^−1^)	0.75 ± 0.34a	2.05 ± 0.05a	2.75 ± 0.05a	1.88 ± 1.48a	2.11 ± 0.37a	1.84 ± 0.15a	2.21 ± 0.13a	0.74 ± 0.23a
Carbon	% (w w^−1^)	90.70 ± 0.35a	44.30 ± 0.41d	35.3 ± 0.15e	88.21 ± 1.78b	43.68 ± 0.75d	73.73 ± 0.14c	42.32 ± 0.32d	25.67 ± 1.41^f^
NH_4_^+^-N	(mg kg^−1^)	4.4 ± 4.7b	2.7 ± 3.1b	ND	3083.5 ± 143.2a	3363.7 ± 335.0a	15.5 ± 3.7b	23.7 ± 22.2b	606.2 ± 16.4b
NO_3_^–^N	(mg kg^−1^)	0.8 ± 0.1b	24.0 ± 2.1b	ND	4.7 ± 0.1b	20.6 ± 2.7b	0.5 ± 0.1b	2.3 ± 1.2b	635.1 ± 15.2a

ND not determined, as KCl extraction of slurry is not an appropriate measure plant-available N.

Letters indicate significant differences between amendments using a one-way anova (p < 0.05; n = 3).

The change in total N in manure and wood biochars following immersion in the slurry was similar to that through NH_3_ exposure, compared to unexposed biochars. However, in both biochars, a much smaller portion of this added N was immediately plant-available after immersion in slurry, 0.0–0.01 g N kg^−1^, compared to after exposure to NH_3_, 0.005–3.4 g N kg^−1^ (Table 1). Thus, sorbed N on biochars following NH_3_ exposure is more plant-available than sorbed N from the slurry.

Manure biochar contained significantly greater total nutrients (acid-digestible) and plant-available (Mehlich III extractable) nutrients by mass than wood biochar, specifically Ca, Mg, and P (Tables 2, 3). This corroborates with previous reports of large amounts of ash minerals in biochars from manure feedstocks compared to woody feedstocks^23,36^. Calcium (Ca) comprised more than 15% of manure biochar mass and less than 1% of wood biochar mass. The high Ca concentration in manure biochar resulted from the regular liming of fresh manure solids after screw-pressing. Significantly greater total micronutrients (B, Cu, Fe, Mn, Zn) were observed in manure biochar compared to wood biochar, 3.14 vs. 0.80 g kg^−1^, respectively. This trend was reversed for extractable elements: wood biochar contained 2.5-fold greater Mehlich III-extractable micronutrients (358.3 mg g^−1^) than manure biochar (146.1 mg g^−1^).

**Table 2:** Total nutrients measured in acid-digested (HClO_4_ + HNO_3_) amendments used for the greenhouse trial ± the standard deviation.

Analysis	Unit	Wood biochar	Manure biochar	Slurry
Al	(g kg^−1^)	0.38 + 0.02b	4.03 + 1.57a	0.35 + 0.28b
Ca	(g kg^−1^)	5.3 + 0.0b	158.1 + 12.4a	49.5 + 42.0a
K	(g kg^−1^)	5.5 + 0.3a	12.8 + 1.9a	37.5 + 32.4a
Mg	(g kg^−1^)	0.75 + 0.01b	15.93 + 0.12a	7.66 + 6.56ab
Na	(g kg^−1^)	0.47 + 0.01a	3.92 + 0.51a	8.78 + 7.55a
P	(g kg^−1^)	0.55 + 0.02b	11.90 + 0.90a	7.17 + 6.16ab
S	(g kg^−1^)	0.09 + 0.00a	2.30 + 0.20a	3.53 + 3.03a
Micronutrients (B + Cu + Fe + Mn + Zn)	(g kg^−1^)	0.80 + 0.03b	3.14 + 0.19a	0.99 + 0.75b
Heavy metals (Cd, Pb)	(mg kg^−1^)	3.04 + 0.33b	15.28 + 3.64a	2.34 + 1.08b

Letters indicate significant differences between amendments using a one-way anova (p < 0.05; n = 3).

**Table 3:** Plant-available nutrients in amendments, extracted with Mehlich III ± the standard deviation.

Analysis	Unit	Wood biochar	Manure biochar	Wood biochar NH_3_ + CO_2_	Manure biochar CO_2_ + NH_3_	Wood biochar + slurry	Manure biochar + slurry
Al	(mg kg^−1^)	130.4 ± 4.6a	0.8 ± 0.5d	83.5 ± 4.0b	0.0 ± 0.1d	17.4 ± 3.6c	10.8 ± 1.6c
Ca	(g kg^−1^)	3.46 ± 0.06e	25.92 ± 0.03a	2.53 ± 0.08f.	21.84 ± 0.16c	11.91 ± 0.36d	23.80 ± 0.33b
K	(g kg^−1^)	7.00 ± 0.05d	9.87 ± 0.14c	6.47 ± 0.07d	10.41 ± 0.20c	18.70 ± 0.82a	13.12 ± 0.31b
Mg	(g kg^−1^)	0.58 ± 0.02d	3.00 ± 0.01a	0.48 ± 0.00d	2.50 ± 0.03c	2.46 ± 0.09c	2.71 ± 0.04b
Na	(g kg^−1^)	0.81 ± 0.06b	2.40 ± 0.01a	0.68 ± 0.04c	2.41 ± 0.00a	2.36 ± 0.00a	2.39 ± 0.00a
P	(g kg^−1^)	0.32 ± 0.00d	1.08 ± 0.01b	0.23 ± 0.00e	0.91 ± 0.00c	1.71 ± 0.06a	0.17 ± 0.03a
S	(mg kg^−1^)	62 ± 2c	302 ± 4a	55 ± 1c	290 ± 3a	214 ± 9b	303 ± 8a
Micronutrients (B, Cu, Fe, Mn, Zn)	(mg kg^−1^)	358 ± 9a	146 ± 1c	260 ± 9b	122 ± 1d	264 ± 1b	143 ± 3c
Heavy metals (Cd, Pb)	(mg kg^−1^)	9.83 ± 3.91a	0.84 ± 0.02b	0.14 ± 0.32b	0.75 ± 0.01b	1.42 ± 0.16b	0.61 ± 0.06b

Letters indicate significant differences between amendments using a one-way anova (p < 0.05; n = 3).

The total and Mehlich-III-extractable and therefore plant-available concentrations of heavy metals in both wood and manure biochars were below the EPA threshold values for biosolids intended for agriculture^40,48^. Manure biochar contained significantly greater total concentrations of heavy metals (Cd, Pb) than wood biochar, 15.3 vs. 3.0 mg kg^−1^ (Table 2). However, Mehlich III-extractable heavy metals were 12-fold greater in wood biochar than manure biochar, reaching 9.8 mg g^−1^ vs. 0.84 mg g^−1^, respectively (Table 3).

**Figure 1:**
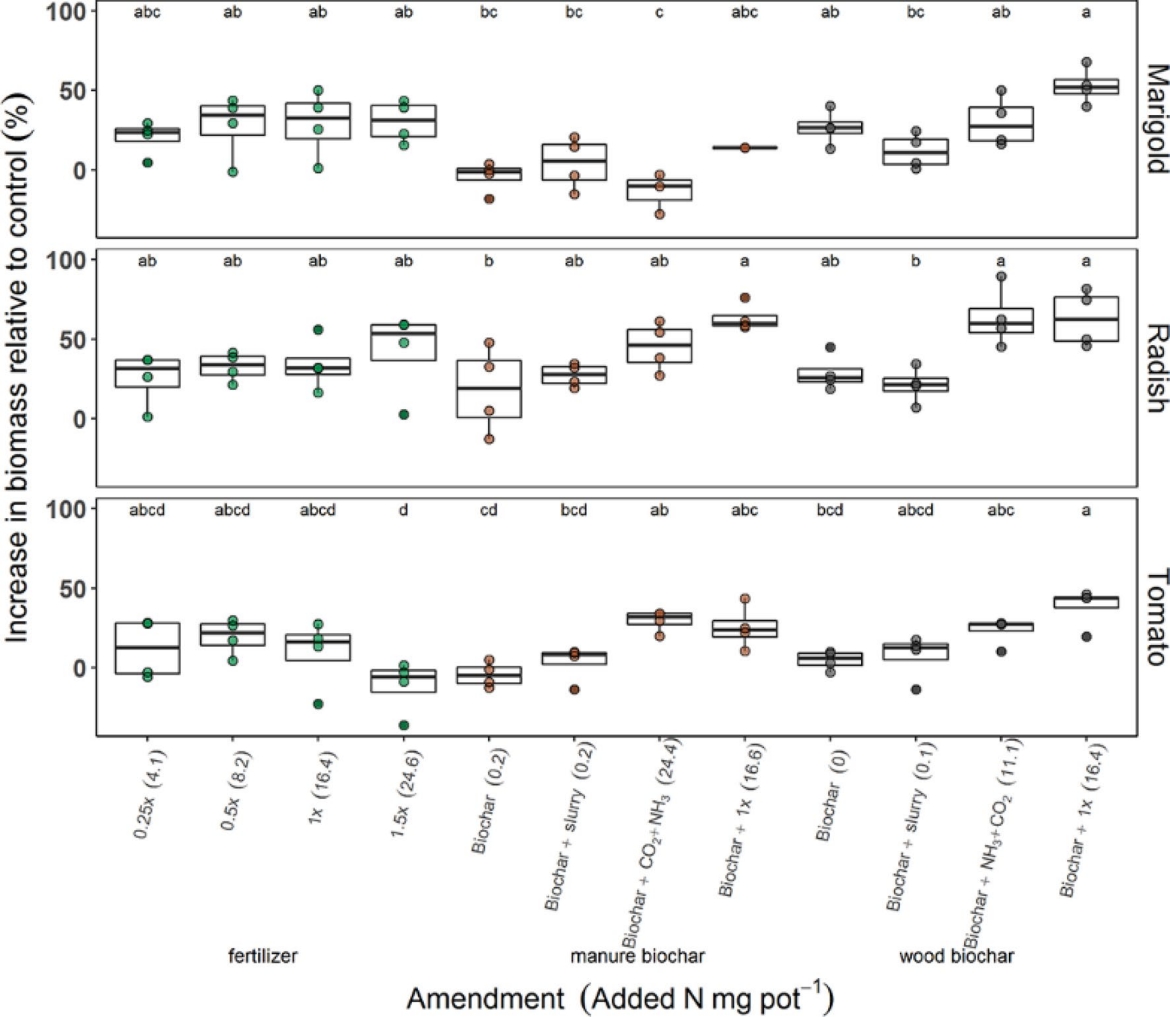
Increase in plant biomass (sum of root and shoot biomass) grown with urea fertilizer (green points), manure biochar (brown points) or wood biochar (gray points) amendments relative to unamended plants (0×). The amount of plant-available N in each type of amendment in parentheses. Letters above the bars indicate significant differences between amendments within plant type (*p* < 0.05; n = 4; whiskers indicate standard errors).

Additions of wood biochar alone increased plant growth (dry weight of above and below-ground biomass) from 4.9 to 29% relative to no additions. Radish plants benefited from additions of manure biochar alone, with 18% greater plant growth than no additions. When urea alone (1×) was added to potting media, plant growth increased by 9–34% relative to unamended plants. Additions of manure or wood biochar together with urea (1×) increased plant growth by 14–63% relative to unamended plants (Fig. 1, Table 4).

**Table 4:** The average ± standard deviation of the pH of potting mix and amendments after 40 days, total plant biomass (shoot and root combined) and relative increase in plant biomass relative to unamended plants (0×), total N plant uptake (shoot and root combined), and relative increase in biomass N uptake in comparison to unamended plants.

Plant	Amendment	pH after 40 days	Plant N uptake (mg pot^−1^)	Relative increase in plant N uptake (% w w^−1^)	Plant biomass (g pot^−1^)	Relative increase in plant biomass (% w w^−1^)
Marigold	0× fert	6.35 ± 0.13cd	35.63 ± 3.87de	NA	2.81 ± 0.36bc	NA
Marigold	0.25× fert	6.28 ± 0.14cd	40.37 ± 7.21bcde	13.28 ± 20.25bcde	3.38 ± 0.31abc	20.27 ± 10.89abc
Marigold	0.5× fert	6.30 ± 0.04cd	38.97 ± 6.45cde	9.36 ± 18.10cde	3.59 ± 0.57ab	27.73 ± 20.15ab
Marigold	1× fert	6.15 ± 0.14d	51.63 ± 4.49abc	44.89 ± 12.61abc	3.63 ± 0.60ab	28.98 ± 21.20ab
Marigold	1.5× fert	6.25 ± 0.11cd	61.36 ± 6.64a	72.20 ± 18.65a	3.66 ± 0.37ab	30.22 ± 13.25ab
Marigold	Manure biochar	7.46 ± 0.16a	31.50 ± 5.24e	− 11.60 ± 14.71e	2.69 ± 0.27bc	− 4.27 ± 9.67bc
Marigold	Manure biochar + slurry	7.35 ± 0.43a	34.10 ± 4.93de	− 4.30 ± 13.83de	2.93 ± 0.46bc	4.00 ± 16.51bc
Marigold	Manure biochar CO_2_ + NH_3_	7.41 ± 0.24a	42.74 ± 4.38bcde	19.95 ± 12.29bcde	2.43 ± 0.36c	− 13.72 ± 12.77c
Marigold	Manure biochar + 1×	7.35 ± 0.04a	52.28 ± 0.00abc	46.71 ± 0.00abc	3.20 ± 0.00abc	13.78 ± 0.00abc
Marigold	Wood biochar	6.53 ± 0.19cd	32.40 ± 2.46e	− 9.08 ± 6.92e	3.56 ± 0.31ab	26.58 ± 10.89ab
Marigold	Wood biochar + slurry	7.03 ± 0.18ab	33.20 ± 1.63de	− 6.84 ± 4.56de	3.14 ± 0.31bc	11.64 ± 11.16bc
Marigold	Wood biochar NH_3_ + CO_2_	6.62 ± 0.13bc	46.44 ± 9.15bcd	30.33 ± 25.67bcd	3.66 ± 0.44ab	30.22 ± 15.82ab
Marigold	Wood biochar + 1×	6.51 ± 0.15cd	53.33 ± 3.45ab	49.66 ± 9.67ab	4.30 ± 0.33a	52.80 ± 11.58a
Radish	0× fert	6.48 ± 0.12cd	29.60 ± 7.33d	NA	3.08 ± 0.27c	NA
Radish	0.25× fert	6.51 ± 0.16cd	40.06 ± 4.41bcd	35.33 ± 14.89bcd	3.87 ± 0.52abc	25.39 ± 16.91ab
Radish	0.5× fert	6.34 ± 0.14d	42.74 ± 7.71abcd	44.37 ± 26.04abcd	4.09 ± 0.28abc	32.69 ± 9.13ab
Radish	1× fert	6.35 ± 0.15d	53.04 ± 6.73ab	79.17 ± 22.74ab	4.13 ± 0.50abc	34.06 ± 16.35ab
Radish	1.5× fert	6.37 ± 0.14d	55.94 ± 8.38a	88.96 ± 28.30a	4.39 ± 0.83ab	42.25 ± 26.80ab
Radish	Manure biochar	7.22 ± 0.10ab	30.19 ± 4.82d	1.99 ± 16.27d	3.65 ± 0.84bc	18.25 ± 27.22b
Radish	Manure biochar + slurry	7.31 ± 0.12a	31.60 ± 1.78d	6.74 ± 6.00d	3.93 ± 0.22abc	27.33 ± 7.19ab
Radish	Manure biochar CO_2_ + NH_3_	7.25 ± 0.13a	55.28 ± 8.68a	86.75 ± 29.34a	4.48 ± 0.48ab	45.26 ± 15.45ab
Radish	Manure biochar + 1×	7.02 ± 0.56ab	46.72 ± 1.03abc	57.82 ± 3.49abc	5.04 ± 0.27a	63.42 ± 8.62a
Radish	Wood biochar	6.74 ± 0.04bcd	31.68 ± 3.45d	7.01 ± 11.64d	3.97 ± 0.35abc	28.79 ± 11.34ab
Radish	Wood biochar + slurry	6.90 ± 0.16abc	32.58 ± 2.55cd	10.05 ± 8.61cd	3.74 ± 0.35bc	21.17 ± 11.28b
Radish	Wood biochar NH_3_ + CO_2_	6.45 ± 0.06cd	50.52 ± 8.56ab	70.65 ± 28.92ab	5.05 ± 0.58a	63.67 ± 18.83a
Radish	Wood biochar + 1×	6.48 ± 0.14cd	48.51 ± 2.66ab	63.88 ± 8.97ab	5.03 ± 0.55a	63.10 ± 17.80a
Tomato	0× fert	6.11 ± 0.07d	39.70 ± 3.52def	NA	3.52 ± 0.43bcd	NA
Tomato	0.25× fert	6.15 ± 0.18d	46.36 ± 4.73cde	16.79 ± 11.92cde	3.93 ± 0.66abcd	11.81 ± 18.76abcd
Tomato	0.5× fert	6.33 ± 0.55cd	52.98 ± 2.68bc	33.46 ± 6.74bc	4.20 ± 0.40abcd	19.49 ± 11.37abcd
Tomato	1× fert	6.17 ± 0.02d	60.72 ± 3.40ab	52.95 ± 8.58ab	3.84 ± 0.78abcd	9.10 ± 22.11abcd
Tomato	1.5× fert	6.36 ± 0.30cd	71.55 ± 11.10a	80.23 ± 27.97a	3.11 ± 0.60d	− 11.59 ± 16.98d
Tomato	Manure biochar	7.08 ± 0.12a	34.26 ± 2.42f	− 13.69 ± 6.09f	3.36 ± 0.28cd	− 4.48 ± 7.89cd
Tomato	Manure biochar + slurry	7.07 ± 0.13a	36.95 ± 1.50ef	− 6.91 ± 3.78ef	3.63 ± 0.40bcd	3.13 ± 11.34bcd
Tomato	Manure biochar CO_2_ + NH_3_	6.83 ± 0.46abc	51.64 ± 4.60bc	30.09 ± 11.58bc	4.55 ± 0.24ab	29.30 ± 6.71ab
Tomato	Manure biochar + 1×	7.05 ± 0.03ab	49.75 ± 3.39bcd	25.32 ± 8.55bcd	4.40 ± 0.48abc	25.18 ± 13.67abc
Tomato	Wood biochar	6.30 ± 0.14cd	35.60 ± 3.47ef	− 10.33 ± 8.74ef	3.68 ± 0.21bcd	4.62 ± 5.89bcd
Tomato	Wood biochar + slurry	6.47 ± 0.12bcd	40.16 ± 2.27def	1.16 ± 5.73def	3.77 ± 0.50abcd	7.18 ± 14.22abcd
Tomato	Wood biochar NH_3_ + CO_2_	6.32 ± 0.11cd	51.79 ± 1.76bc	30.45 ± 4.42bc	4.33 ± 0.31abc	23.19 ± 8.73abc
Tomato	Wood biochar + 1×	6.25 ± 0.05cd	57.20 ± 4.41bc	44.10 ± 11.11bc	4.86 ± 0.44a	38.26 ± 12.56a
Marigold	0× fert	6.35 ± 0.13cd [ghij]	35.63 ± 3.87de [ghijklm]	NA	2.81 ± 0.36bc [hij]	NA
Marigold	0.25× fert	6.28 ± 0.14cd [hij]	40.37 ± 7.21bcde [efghijklm]	13.28 ± 20.25bcde [ghijklm]	3.38 ± 0.31abc [defghij]	20.27 ± 10.89abc [bcdefg]
Marigold	0.5× fert	6.30 ± 0.04cd [hij]	38.97 ± 6.45cde [efghijklm]	9.36 ± 18.10cde [ghijklm]	3.59 ± 0.57ab [cdefghij]	27.73 ± 20.15ab [abcdefg]
Marigold	1× fert	6.15 ± 0.14d [j]	51.63 ± 4.49abc [bcdef]	44.89 ± 12.61abc [bcdefghi]	3.63 ± 0.60ab [cdefghij]	28.98 ± 21.20ab [abcdefg]
Marigold	1.5× fert	6.25 ± 0.11cd [hij]	61.36 ± 6.64a [ab]	72.20 ± 18.65a [abcd]	3.66 ± 0.37ab [cdefghij]	30.22 ± 13.25ab [abcdef]
Marigold	Manure biochar	7.46 ± 0.16a []	31.50 ± 5.24e [m]	− 11.60 ± 14.71e [lm]	2.69 ± 0.27bc [ij]	− 4.27 ± 9.67bc [efg]
Marigold	Manure biochar + slurry	7.35 ± 0.43a []	34.10 ± 4.93de [ijklm]	− 4.30 ± 13.83de [jklm]	2.93 ± 0.46bc [ghij]	4.00 ± 16.51bc [cdefg]
Marigold	Manure biochar CO_2_ + NH_3_	7.41 ± 0.24a []	42.74 ± 4.38bcde [defghijklm]	19.95 ± 12.29bcde [efghijklm]	2.43 ± 0.36c [j]	− 13.72 ± 12.77c [fg]
Marigold	Manure biochar + 1×	7.35 ± 0.04a []	52.28 ± 0.00abc [bcdefgh]	46.71 ± 0.00abc [abcdefghij]	3.20 ± 0.00abc [defghij]	13.78 ± 0.00abc [abcdefg]
Marigold	Wood biochar	6.53 ± 0.19cd []	32.40 ± 2.46e [jklm]	− 9.08 ± 6.92e [klm]	3.56 ± 0.31ab [defghij]	26.58 ± 10.89ab [abcdefg]
Marigold	Wood biochar + slurry	7.03 ± 0.18ab []	33.20 ± 1.63de [jklm]	− 6.84 ± 4.56de [jklm]	3.14 ± 0.31bc [efghij]	11.64 ± 11.16bc [bcdefg]
Marigold	Wood biochar NH_3_ + CO_2_	6.62 ± 0.13bc []	46.44 ± 9.15bcd [cdefghijk]	30.33 ± 25.67bcd [defghijkl]	3.66 ± 0.44ab [cdefghij]	30.22 ± 15.82ab [abcdef]
Marigold	Wood biochar + 1×	6.51 ± 0.15cd []	53.33 ± 3.45ab [bcde]	49.66 ± 9.67ab [abcdefgh]	4.30 ± 0.33a [abcdef]	52.80 ± 11.58a [ab]
Radish	0× fert	6.48 ± 0.12cd []	29.60 ± 7.33d [m]	NA	3.08 ± 0.27c [fghij]	NA
Radish	0.25× fert	6.51 ± 0.16cd []	40.06 ± 4.41bcd [efghijklm]	35.33 ± 14.89bcd [cdefghij]	3.87 ± 0.52abc [abcdefghi]	25.39 ± 16.91ab [abcdefg]
Radish	0.5× fert	6.34 ± 0.14d []	42.74 ± 7.71abcd [defghijklm]	44.37 ± 26.04abcd [bcdefghi]	4.09 ± 0.28abc [abcdefgh]	32.69 ± 9.13ab [abcde]
Radish	1× fert	6.35 ± 0.15d []	53.04 ± 6.73ab [bcde]	79.17 ± 22.74ab [abc]	4.13 ± 0.50abc [abcdefg]	34.06 ± 16.35ab [abcde]
Radish	1.5× fert	6.37 ± 0.14d []	55.94 ± 8.38a [bcd]	88.96 ± 28.30a [a]	4.39 ± 0.83ab [abcde]	42.25 ± 26.80ab [abcd]
Radish	Manure biochar	7.22 ± 0.10ab []	30.19 ± 4.82d [m]	1.99 ± 16.27d [ijklm]	3.65 ± 0.84bc [cdefghij]	18.25 ± 27.22b [bcdefg]
Radish	Manure biochar + slurry	7.31 ± 0.12a []	31.60 ± 1.78d [lm]	6.74 ± 6.00d [hijklm]	3.93 ± 0.22abc [abcdefghi]	27.33 ± 7.19ab [abcdefg]
Radish	Manure biochar CO_2_ + NH_3_	7.25 ± 0.13a []	55.28 ± 8.68a [bcd]	86.75 ± 29.34a [ab]	4.48 ± 0.48ab [abcd]	45.26 ± 15.45ab [abc]
Radish	Manure biochar + 1×	7.02 ± 0.56ab []	46.72 ± 1.03abc [bcdefghij]	57.82 ± 3.49abc [abcdef]	5.04 ± 0.27a [a]	63.42 ± 8.62a [a]
Radish	Wood biochar	6.74 ± 0.04bcd []	31.68 ± 3.45d [klm]	7.01 ± 11.64d [hijklm]	3.97 ± 0.35abc [abcdefghi]	28.79 ± 11.34ab [abcdefg]
Radish	Wood biochar + slurry	6.90 ± 0.16abc []	32.58 ± 2.55cd [jklm]	10.05 ± 8.61cd [ghijklm]	3.74 ± 0.35bc [bcdefghij]	21.17 ± 11.28b [bcdefg]
Radish	Wood biochar NH_3_ + CO_2_	6.45 ± 0.06cd []	50.52 ± 8.56ab [bcdef]	70.65 ± 28.92ab [abcd]	5.05 ± 0.58a [a]	63.67 ± 18.83a [a]
Radish	Wood biochar + 1×	6.48 ± 0.14cd []	48.51 ± 2.66ab [bcdefghi]	63.88 ± 8.97ab [abcde]	5.03 ± 0.55a [ab]	63.10 ± 17.80a [a]
Tomato	0× fert	6.11 ± 0.07d []	39.70 ± 3.52def [efghijklm]	NA	3.52 ± 0.43bcd [defghij]	NA
Tomato	0.25× fert	6.15 ± 0.18d []	46.36 ± 4.73cde [cdefghijkl]	16.79 ± 11.92cde [fghijklm]	3.93 ± 0.66abcd [abcdefghi]	11.81 ± 18.76abcd [bcdefg]
Tomato	0.5× fert	6.33 ± 0.55cd []	52.98 ± 2.68bc [bcde]	33.46 ± 6.74bc [defghijk]	4.20 ± 0.40abcd [abcdefg]	19.49 ± 11.37abcd [bcdefg]
Tomato	1× fert	6.17 ± 0.02d []	60.72 ± 3.40ab [abc]	52.95 ± 8.58ab [abcdefg]	3.84 ± 0.78abcd [abcdefghi]	9.10 ± 22.11abcd [cdefg]
Tomato	1.5× fert	6.36 ± 0.30cd []	71.55 ± 11.10a [a]	80.23 ± 27.97a [ab]	3.11 ± 0.60d [efghij]	− 11.59 ± 16.98d [g]
Tomato	Manure biochar	7.08 ± 0.12a []	34.26 ± 2.42f [hijklm]	− 13.69 ± 6.09f [m]	3.36 ± 0.28cd [defghij]	− 4.48 ± 7.89cd [efg]
Tomato	Manure biochar + slurry	7.07 ± 0.13a []	36.95 ± 1.50ef [fghijklm]	− 6.91 ± 3.78ef [jklm]	3.63 ± 0.40bcd [cdefghij]	3.13 ± 11.34bcd [defg]
Tomato	Manure biochar CO_2_ + NH_3_	6.83 ± 0.46abc []	51.64 ± 4.60bc [bcdef]	30.09 ± 11.58bc [defghijklm]	4.55 ± 0.24ab [abcd]	29.30 ± 6.71ab [abcdefg]
Tomato	Manure biochar + 1×	7.05 ± 0.03ab []	49.75 ± 3.39bcd [bcdefg]	25.32 ± 8.55bcd [efghijklm]	4.40 ± 0.48abc [abcde]	25.18 ± 13.67abc [abcdefg]
Tomato	Wood biochar	6.30 ± 0.14cd []	35.60 ± 3.47ef [ghijklm]	− 10.33 ± 8.74ef [klm]	3.68 ± 0.21bcd [cdefghij]	4.62 ± 5.89bcd [cdefg]
Tomato	Wood biochar + slurry	6.47 ± 0.12bcd []	40.16 ± 2.27def [efghijklm]	1.16 ± 5.73def [ijklm]	3.77 ± 0.50abcd [abcdefghij]	7.18 ± 14.22abcd [cdefg]
Tomato	Wood biochar NH_3_ + CO_2_	6.32 ± 0.11cd []	51.79 ± 1.76bc [bcde]	30.45 ± 4.42bc [defghijkl]	4.33 ± 0.31abc [abcdef]	23.19 ± 8.73abc [abcdefg]
Tomato	Wood biochar + 1×	6.25 ± 0.05cd []	57.20 ± 4.41bc [abcd]	44.10 ± 11.11bc [bcdefghi]	4.86 ± 0.44a [abc]	38.26 ± 12.56a [abcd]

Letters indicate significant differences from a one-way anova between amendments within plant type (p < 0.05; n = 4; results for a two-way anova include in supplementary online material Table SI 5).

Different forms of N added with biochars had different effects on plant growth. Wood biochar applied together with urea (1×) promoted 0–18% greater plant growth than wood biochar treated with NH_3_ despite the fact that the total N content in both types of amendments was identical, 1.88%. Furthermore, wood biochar and urea promoted 29–37% more plant growth compared to slurry-immersed biochar despite the similar total N values between the two types of amendments, 1.88% vs 1.84% (Tables 1, 4).

Differences in plant growth with the type of biochar and added N were also apparent. Overall, plant growth was 6–34% lower after adding manure biochar exposed to NH_3_ compared to adding wood biochar exposed to NH_3_. These effects of adding manure biochar exposed to NH_3_ on plant growth varied between plant type. In a one-way anova of the effect of amendment type on plant growth, between plant types, we observed that supplementing manure biochar with N by exposing it to NH_3_ significantly improved tomato growth above tomatoes grown with only manure biochar, by 35%. Supplementing manure biochar with urea fertilizer significantly improved radish growth by 38% compared to additions of manure biochar alone (Fig. 1, Table 4).

Using a two-way anova of the effect of amendments and plant types on growth, plant growth in radishes amended with manure or wood biochar added with urea fertilizer and wood biochar treated with NH_3_ were significantly higher compared to the growth of all plant types grown with manure biochar alone. Furthermore, no significant differences in plant growth were apparent between radish and tomato amended with manure or wood biochars treated with NH_3_ or added with urea fertilizer, compared to adding urea alone, in any amount (Fig. SI 1, Table SI 5). Thus, supplementing wood or manure biochar with N from NH_3_ exposure or urea fertilizer proved just as good for plants as adding urea fertilizer alone.

Germination was affected by plant type and amendment, and was lower with greater nutrient additions for marigold and radish, and higher with the highest nutrient additions for tomato. Unamended and manure biochar-amended marigold and radish had the highest germination rates, 80–90%, while tomato plants amended with the highest urea application rates, 1.5×, and wood biochar, reached 100% germination (Table SI 4).

After 40 days, the pH of pots amended with manure biochar (7.02–7.46) was significantly higher than the potting mix amended with urea (1×) or the unamended potting mix (6.11–6.48), based on both a one-way anova of the effect of amendment type on pH (Table 4) and a two-way anova of the effect of amendment type and plant type on pH (Table SI 5). The pH of pots with wood biochar immersed in the liquid manure (6.47–7.03) was highest among all wood biochar treatments (6.25–6.74). In contrast, wood biochar alone did not significantly increase the pH of the potting mix, relative to the unamended pots (Table 4, Table SI 5).

**Figure 2:**
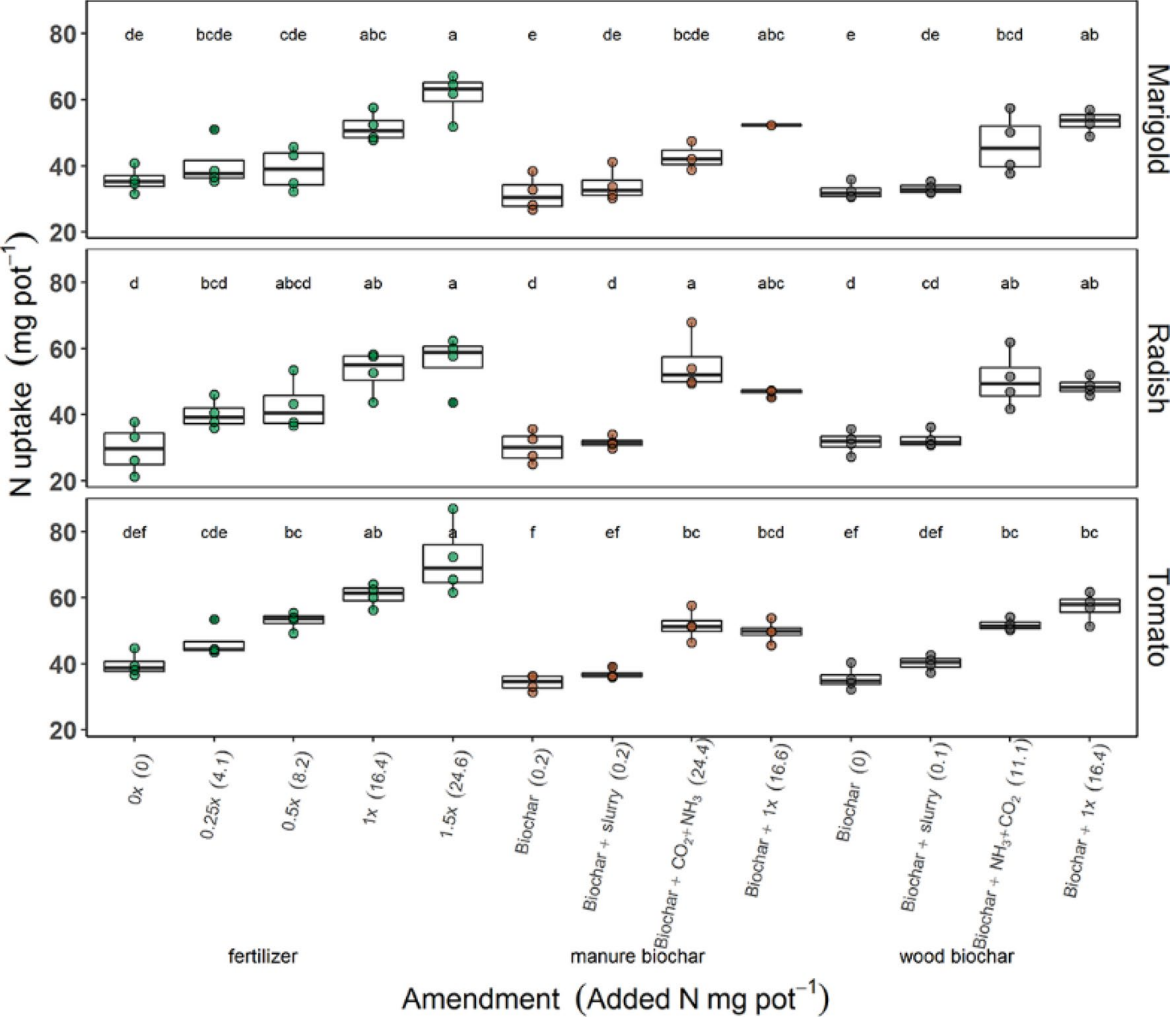
Total nitrogen uptake in shoot and root biomass of plants grown with urea fertilizer, manure biochar or wood biochar amendments or no amendments (0×). Letters above the bars indicate significant differences between amendments within plant type (*p* < 0.05; n = 4).

**Figure 3:**
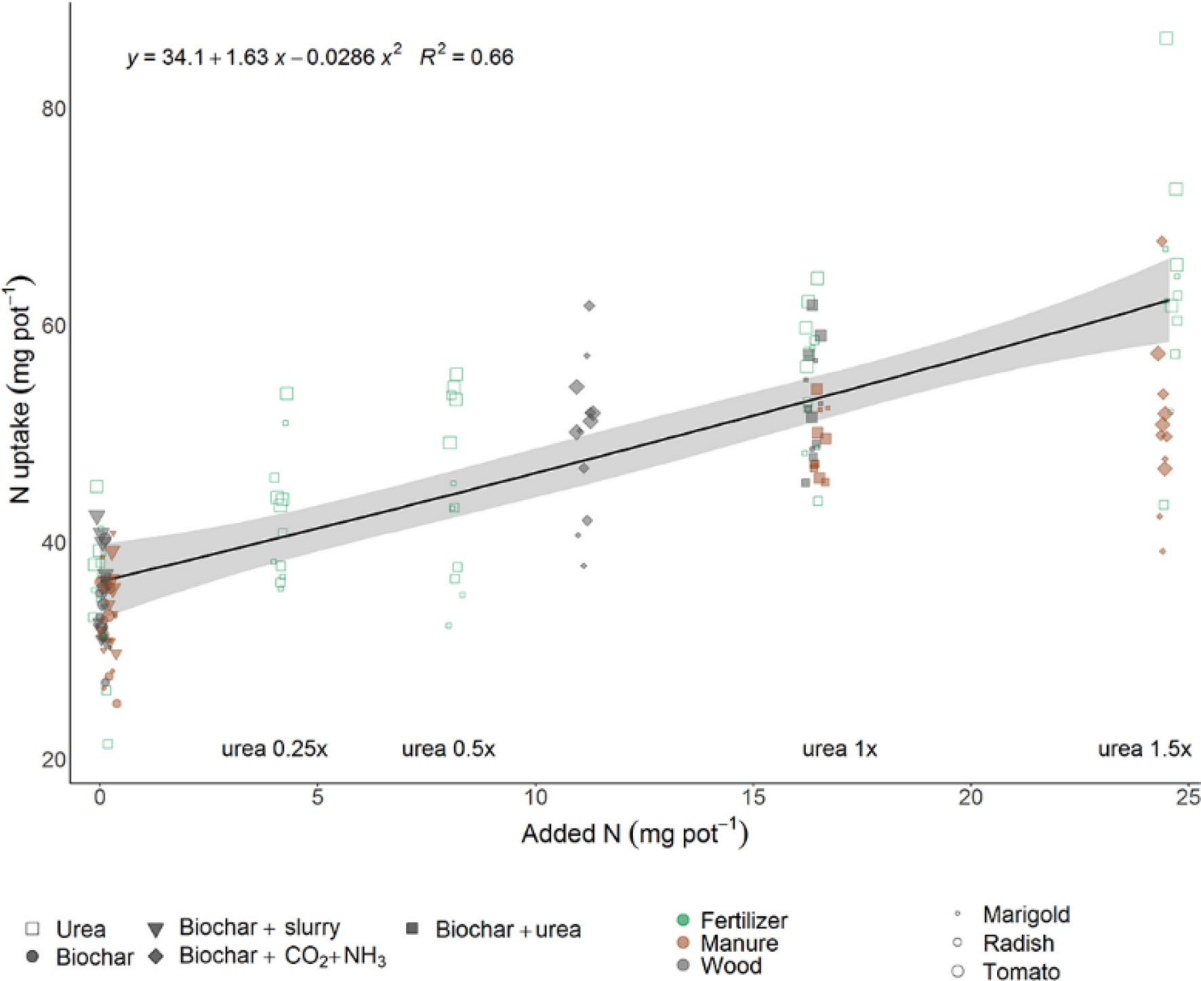
Nitrogen uptake of shoots and roots of plants grown with amendments varying in initial N content (points). “Added N” refers to the KCl-extractable N of all amendments, with the assumption that all urea-N is KCl-extractable. A linear regression was conducted for urea-based amendments (urea, biochar + urea). The 95% confidence interval is shown by the gray shaded line, and the R^2^ value for the quadratic equation is presented (n = 4).

Plant N uptake increased with increasing N additions (Fig. 2). The range of urea additions encompassed N additions of all other amendments (Fig. 3) indicating positive growth responses across all rates of added N. Nitrogen from wood biochar exposed to NH_3_ appears to be as plant available as urea 1×, since N uptake was slightly higher per unit N added (Fig. 3). Furthermore, the N uptake of plants grown with wood biochar treated with NH_3_ was not significantly different than the N uptake of plants grown with urea 1×, based on either the one-way anova of the effect of amendment on N uptake, between plant types or the two-way anova evaluating N uptake as affected by both amendment and plant type (Fig. 2, Table 4, Fig. SI 2, Table SI 5). Nitrogen from manure biochar exposed to NH_3_ appears to be less plant available than urea, since N uptake was smaller per unit N added (Fig. 3), although these results varied between plant type. Radish plants grown with manure biochar treated with NH_3_ had similar N uptake as when grown with the highest urea treatment.

When added with urea, biochar did not increase N uptake, compared to N uptake with urea additions alone. However, adsorption of NH_3_ onto biochars provided both immediately-available plant-available N and longer-term mineralizable N, allowing for greater plant uptake than equivalent amendments of urea. The initial KCl-extractable N in manure biochar exposed to NH_3_ was comparable to urea 0.5× additions, indicating the mineralizability of N incorporated through NH_3_-exposure of manure biochar (Fig. 3). The difference in total N in wood biochar following NH_3_ exposure was equal to the amount of N in urea 1× addition, yet the KCl-extractable N was comparable to the amount of N in urea 0.25×. Nevertheless, compared to urea 0.25×, plant N uptake in wood biochar sorbed with NH_3_ was 12–26% greater. Moreover, in all experiments, NH_3_-exposed biochars outperformed biochars immersed in the liquid fraction, increasing N uptake by 29–55% for wood biochar and 25–75% for manure.

## Discussion

The feedstock and pyrolysis conditions used to produce biochar for these experiments did not result in phototoxicity, as no difference in germination was detectable between plants with or without biochar. The moderately high temperatures used for pyrolysis, 500 °C may have reduced the phototoxicity reported from lower-temperature biochars^49^. The greater increase in KCl-extractable N than in total N in manure biochar exposed to NH_3_ may be related to CO_2_ exposure that increased extractability of the N already present in the biochar. Yet it is unclear whether CO_2_ can solubilize trapped inorganic N species or cleave bonds of organic N. Either way, all N from NH_3_ added to manure biochar was KCl-extractable. On the other hand, the increase in total N in wood biochar exposed to NH_3_ was 19-fold greater than manure biochar exposed to NH_3_, even though both materials contained the same amount of KCl-extractable N. This means that the majority of N in wood biochar treated with NH_3_ more strongly interacted with the biochar than the NH_3_-derived N in manure biochar, in alignment with previous studies^29,40^ and was therefore not plant-available.

Capturing volatile NH_3_ from the liquid fraction of dairy waste onto sorbents such as biochars was more successful for recycling nutrients for use as potting media than immersing sorbents in the liquid fraction. Plant N uptake was 25–75% and 29–55% greater in manure and wood biochars, respectively, for the NH_3_ treatment compared to slurry immersion. Lower plant growth and N uptake with biochar treated with the slurry could be attributed to a combination of both the lower amount of readily plant-available N (i.e., KCl-extractable) and lower rates of mineralization of organic N. The plant-availability of organic N in the liquid fraction is time-dependent and varies with manure age and processing. Pettygrove et al.^50^ determined that 27–44% of total N in fresh lagoon slurry was mineralized after 6 weeks when incubated in a sandy loam soil. The low pH of our peat potting media may have slowed microbial mineralization rates of slurry N compared to rates reported for mineral soils^51^.

Delivering N from NH_3_-enriched manure biochar to plants was just as effective as adding urea with manure biochar. The increase in plant N uptake relative to the control was 43–55% vs. 47–52%, respectively. The high plant-availability of N in manure biochar doped with N may indicate that an NH_4_^+^ salt such as NH_4_HCO_3_ formed from repeated exposure to NH_3_ and CO_2_, preserving all sorbed N in a plant-available form, as observed with CO_2_ captured within NH_3_ solvents^52–54^. Mineral forms of N were detected in human manure biochars and wood biochars exposed to repeated intervals of NH_3_ and CO_2_ using XPS, although two-fold more electrostatically-sorbed NH_4_^+^ was detected in NH_3_- and CO_2_-exposed wood biochar compared to NH_3_- and CO_2_-exposed manure biochar^40^.

Using median international prices for fertilizer nutrients, the value of extractable N in biochars exposed to NH_3_ is 1.7 USD Mg^−1^ for manure biochar and 1.5 USD Mg^−1^ for wood biochar^39^. The benefit of plant-extractable minerals (Mehlich-III extractable) in dairy manure biochar on plant growth was not evaluated experimentally, since equivalent extractable nutrients in manure biochar were added to all other treatments. Nevertheless, we can estimate the added monetary value of plant-extractable P and K in manure biochar, together with plant-available N, as 15.4 USD Mg^−1^ for manure biochar exposed to NH_3_ and 9.5 USD Mg^−1^ for wood biochar exposed to NH_3_ using literature values^39^.

The increase in plant N uptake with additions of manure biochar exposed to NH_3_ relative to unamended plants reached 20–87% or 7.1–25.7 mg N pot^−1^, which, accounting for the amount of biochar added to each pot, is equivalent to 1–3.6 kg N Mg^−1^ manure biochar. If we account for more than 624,000 dairy cows in NY State^55^, each generating approximately 18.8 Mg (dry) manure year^−1^^56^, this amount of available N in NH_3_-exposed manure biochar scales to 11,732–42,232 Mg N year^−1^ or 6–21.5 million USD year^−1^, based on the average price of N fertilizer (0.51 USD kg^−1^^39^). With approximately 51,360 Mg N applied to grain corn in NY State in 2018^4,6^, separated dairy manure treated with NH_3_ can offset 23–82% of N fertilizer needs while stabilizing both the solid and liquid fraction of manure for addressing both environmental pollution as well as recycling N to agriculture.

A novel fertilizer has been developed from N-rich dairy waste which performs equally well compared to conventional urea fertilizer per unit applied N. Not only did NH_3_-sorbed wood biochar contain similar amounts of plant-available N as NH_3_-manure biochar, but promoted greater plant biomass growth and plant-N uptake than manure biochar or conventional urea fertilizer. Despite the greater NH_3_-derived N enrichment in wood than manure biochar, the precipitation of NH_3_-salts on manure biochar in comparison to chemisorption of NH_3_ to wood, points at manure biochar being a more efficient approach to recycle manure N as a fertilizer.

This research demonstrates that it is possible to convert dairy manure solids into a biochar that can adsorb volatile NH_3_ for use as a N fertilizer. Conversion of dairy manures with high water contents into a dry and N-rich soil amendment with N use efficiency by plants commensurate with urea fertilizer may provide life-cycle benefits to water quality and greenhouse gas emissions that should be studied in the future. Future studies should include scaling up CO_2_ and NH_3_ exposure of biochar. Multi-year field studies with crops such as corn should examine the long-term availability of N-doped biochars and potential differences in leaching and gaseous N losses. The feasibility of cost-effectively operating dairy manure pyrolysis as well as adsorption of N to biochars should be studied on farms and by small industry. Techno-economic studies should quantify the ability to optimize the production and distribution of such fertilizers at different scales and under different economic conditions.

## Materials and methods

### Enriching biochar amendments with nitrogen

We evaluated the effect of N-enriched dairy manure biochar and N-enriched wood biochar on plant growth. The first type of biochar was created from anaerobically-digested dairy manure solids (‘solid fraction’), screw-pressed at a dairy farm in upstate New York in April 2018. The solid fraction was charred in a modified muffle furnace with a rotating paddle at 500 °C for 30 min. The liquid fraction of the screw-pressed dairy manure was also collected, sieved with a 425 μm mesh sieve to remove solids, and stored at − 4 °C. The second type of biochar made from Douglas fir wood (*Pseudotsuga menziesii*) using high-temperature gasification, was provided by Green Tree Garden Supply (Ithaca, NY). Both biochars were sieved to below 2 mm particle size.

Manure biochar and wood biochar were enriched with N through two methods: (1) repeated, sequential exposure to CO_2_ and NH_3_, or (2) immersion in the sieved liquid fraction ('liquid fraction'), which contained a mixture of N species. The effect of these two N-enriched amendments on plant growth was compared to (3) separate additions of each biochar (manure or wood biochar) with urea fertilizer, (4) urea additions without biochar, (5) separate additions of each biochar without urea fertilizer, or (6) no additions of biochar or urea.

For the first N-enrichment method, 200 g of biochar were loaded in a 4-L Buechner funnel suspended upright inside a drying oven at 30 °C. The bottom of the funnel was connected to gas flow via silicone tubing, and the top covered with a lid and with parafilm. Manure biochar was first exposed to CO_2_ gas (Instrument grade, Airgas, Ithaca, NY) for one hour. After one hour, CO_2_ flow ceased, and NH_3_ flow commenced for one hour. Ammonia gas was generated by pumping air at 4.72 × 10^–4^ m^3^ s^−1^ through a sealed Erlenmeyer flask containing 1 L of 2 M NH_4_OH (pH 12.43) kept on a hot plate at 30 °C. This process was repeated three times (manure biochar CO_2_ + NH_3_). To enrich wood biochar with N, we reversed the order of gas exposure, first NH_3_ then CO_2_, also for three exposure intervals (wood biochar NH_3_ + CO_2_).

For the second N-enrichment method, biochars were immersed in the liquid fraction for 1.5 h at a ratio of 143 g:1 L. The biochar-slurry suspension was contained in a large glass beaker on a hot plate at 30 °C. After the immersion period, the suspension was sieved through a 425-μm mesh sieve to remove residual liquid. Biochars were dried at 80 °C for 2 days. We did not expose slurry-treated biochars to CO_2_.

Duplicate sets of each biochar-N mixture were homogenized and stored in sealed glass jars. The four biochar treatments evaluated were: (1) manure biochar treated with CO_2_ + NH_3_; (2) wood biochar treated with NH_3_ + CO_2_; (3) dairy manure biochar immersed in the liquid fraction; and (4) wood biochar immersed in the liquid fraction. To simplify notation, when describing biochars treated with CO_2_ + NH_3_ (manure) or NH_3_ + CO_2_ (wood), we will refer to NH_3_ exposure without referring to CO_2_, as NH_3_ uptake was our focus.

### Chemical analysis of amendments

Subsamples of amendments were milled and analyzed for total C and N by dry combustion (Elementar; vario EL cube, Langenselbold, Germany). Non-milled amendment subsamples were extracted with 2 M KCl at a ratio of 0.1 g biochar mL^−1^ KCl and tested for NH_4_^+^ and NO_3_^−^ through a colorimetric method on an auto-flow analyzer (AA3 HR AutoAnalyzer, Seal Analytical, Mequon, WI).

Non-milled amendment samples were also analyzed for plant-available and total elements. Plant-available nutrients were extracted using a Mehlich III solution at a ratio of 0.1 g mL^−1^. Total elemental analysis was conducted on 0.5 g of unmilled biochar spiked with 0.25 mg L^−1^ yttrium as an internal standard. Samples were dissolved in a mixture of 30% perchloric acid in nitric acid (70%) at 180 °C. Both Mehlich III extracts and digestate solutions were analyzed by inductively-coupled plasma optical emission spectroscopy (ICP-OES; Spectro Arcos, Ametek Materials Analysis, Kleve, Germany).

### Greenhouse trial to evaluate amendments

A six-week growth trial was conducted to test amendment performance. Three types of plants, either marigold, radish, and tomato, were grown in a peat potting mix (TH6, Theriault and Hachey Peat Moss Ltd., Baie Sainte-Anne, New Brunswick Canada) in square pots 0.3 L by volume to which biochars were added. Seeds were obtained from commercial companies (radish and tomato from Burpee and marigold from Park Seed) and its use complies with relevant institutional, national, and international guidelines and legislation. Wood biochar amendments were added at 10% bulk volume of the square pots, while manure biochar amendments were added at an equivalent C amount as wood biochar. This resulted in unequal mass and nutrients additions (other than C) between wood biochar and manure biochar. To correct for the increased non-N nutrient addition from manure biochar a mixture of dry nutrients was added to all non-manure biochar treatments based on the Mehlich III extractable nutrient content of manure biochar (Tables SI 1, Table SI 2).

We tested the effect of urea additions to compare the effect of N source on plant growth (Table SI 2). Nitrogen sources included biochars enriched in N through exposure to NH_3_, biochars enriched in N through immersion into the liquid fraction, and urea fertilizer additions. Urea additions were adjusted to equal the increase in total N on wood biochar after sequential exposure to NH_3_ and CO_2_ (Table SI 3), 4.9 g N kg biochar^−1^. We did not add slurry alone, as we focused on adding nutrients and fertilizers as dry materials.

For each plant type, five seeds were planted in each pot after filling with the respective media and nutrient additions. Seedlings were thinned to a single seedling after two weeks, and the germination rate recorded for each pot. Pots in which no seeds germinated received seedlings from replicate pots of the same treatment in which more than one seed germinated.

The N equivalency of the N added with the biochar was quantified in comparison to an N-response curve measured from plants grown with urea fertilizer but without biochar (Urea 99% reagent grade, Sigma Aldrich). Five urea application rates were tested based on the increase in total N on wood biochar + NH_3_ (Table 1, Table SI 3): (1) 1.5 times the N increase of wood biochar after NH_3_ and CO_2_ exposure (1.5×) = 52.65 mg N pot^−1^ or 176.8 kg N ha^−1^, (2) the equivalent N-application rate as the N increase of wood biochar (1×) = 35.10 mg N pot^−1^ or 117.9 kg N ha^−1^, (3) half of the N increase (0.5×) = 17.55 mg N pot^−1^ or 58.95 kg N ha^−1^, (4) one quarter of the N increase (0.25×) = 8.78 mg N pot^−1^ or 29.47 kg N ha^−1^, and (5) no added fertilizer (0×). The urea 1 × addition is in the range of the suggested N application for corn production in New York State (78–146 kg N ha^−−1^ without sod history, legume mixture or manure additions), which is highly variable with soil type and with cropping history^57^. As mentioned above, we also tested the plant effect of each biochar added with urea (1×) (Table SI 3).

All plants were irrigated daily with reverse osmosis water to 90% of field capacity, determined gravimetrically. The field capacity of TH6 media amended with wood biochar or manure biochar was calculated as the amount of water remaining in a 0.3 L PVC cylinder filled with soil after saturation and draining overnight. Irrigation was lowered from field capacity by 10% to prevent leaching during the experiment.

To overcome the hydrophobicity of the potting mixtures, pots were initially placed in trays of water to moisten them from the bottom-up. From the second day of the experiment until day 12, pots were misted from the top daily. After germination on day 12, pots were weighed to determine the amount of water needed to reach 90% of field capacity. A description of the thirteen potting mixtures and irrigation amounts is provided in the supplementary information (Table SI 2).

Plants were harvested after 40 days, and wet shoot and root biomass recorded. Shoots were cut at the soil surface, and roots were excavated from each pot. Roots were isolated via washing and sieving. Dry root and shoot biomass was determined after drying at 65 °C for 3 days. The potting mix was dried at 105 °C for 3 days. Total C and N contents in shoots and roots were determined by dry combustion. After the experiment, all potting mixtures were extracted with Mehlich III to determine changes in plant-available nutrients. The pH of potting mixtures was measured before and after the growth trial in 1 g potting mixture in 20 mL deionized water.

For each of three plant types (marigold = *i*_1_, tomato = *i*_2_, radish = *i*_*3*_) and biomass type (roots = *j*_1_, shoots = *j*_2_), the proportional increase in biomass and N uptake across four replicates (*k* = 1:4) of amended plants relative to the average of four replicates of unamended plants for the same plant and biomass type was calculated as follows:

$$Proportion\;of\;biomass\;\left( {N\;uptake} \right)\;increase_{{i,j,k}} = \frac{{biomass\;\left( {N\;uptake} \right)\;amended\;plants_{{i.j.k}} - biomass\;\left( {N\;uptake} \right)\;unamended\;plants_{{i,j,k}} }}{{biomass\;\left( {N\;uptake} \right)\;unamended\;plants_{{i,j}} }} \times 100\;(\% )$$

The N-fertilizer equivalency of amendments was calculated based on the difference in N uptake of plants grown with urea alone (1×) and plants grown with biochar amendments. Using market prices for mineral fertilizers^39^, we also calculated the replacement value of the plant-available N, P, and K in amendments.

### Statistics

Data analysis was carried out in RStudio^58^ and graphs created using *ggplot2*^59^. Least squares of treatment means (LS means) were calculated using *emmeans*^60^. Order-independent *p* values determined with the Student-*t* test were adjusted using Tukey’s method for comparing a family of five estimates at the α = 0.05 threshold. Compact letter displays of pairwise comparisons for a significance level of *p* < 0.05 were created using *multcompView*^61^. Type I analysis of variance (ANOVA) was calculated using an order-dependent *F* test within the *emmeans* package*.* We present a one-way anova of the effect of amendment types on plant growth (dry weight) and plant N uptake in the main manuscript, and include both one-way anova and two-way anova evaluating the effect of amendment type and plant type, and the interaction between them, on plant growth (dry weight) and N uptake in the supplementary material. All mention of ‘significant differences’ refers to the probability of observing an *F* ratio greater than 0.05 given the null hypothesis, Pr(> *F*), or a *p* value < 0.05.

## Supplementary Information

Supplementary Information.

## References

[1] NYSDAM (New York State Department of Agriculture and Markets) New York State Dairy Statistics 2017 annual summary. *NYS Agricultural Statistics Service.* https://agriculture.ny.gov/system/files/documents/2019/06/NYSAnnStat2017.pdf (Department of Agriculture and Markets, Division of Milk Control and DairyServices, 2017).

[2] Lander, C. H., Moffitt, D. & Alt, K. Nutrients available from livestock manure relative to crop growth requirements; Appendix II- manure characteristics. https://www.nrcs.usda.gov/wps/portal/nrcs/detail/national/technical/nra/nri/results/?cid=nrcs143_014175 (United States Department of Agriculture (USDA) Natural Resource Conservation Service (NRCS), 1998).

[3] Magdoff, F. R. & van Es, H. M. (eds.) Building soils for better crops: Sustainable soilmanagement, 3rd edn. Handbook Series 10. (Sustainable Agriculture Research and Extension, College Park, MD, 2009).

[4] Mosheim R (2019). Fertilizer Use and Price.

[5] Wightman JL, Woodbury PB (2016). New York dairy manure management greenhouse gas emissions and mitigation costs (1992–2022). J. Environ. Qual..

[6] USDA NASS (US Department of Agriculture, National Agricultural StatisticsService). 2014–2015 Agricultural Statistics Annual Bulletin, New York. https://www.nass.usda.gov/Statistics_by_State/New_York/Publications/Annual_Statistical_Bulletin/2015/2014-2015%20NY%20Annual%20Bulletin.pdf (New York Agricultural Statistics USDA, National Agricultural Statistics Service, 2014).

[7] Scharf PC, Kitchen NR, Sudduth KA, Davis JG (2006). Spatially variable corn yield is a weak predictor of optimal nitrogen rate. Soil Sci. Soc. Am. J..

[8] Shepherd, T. Karszes, J. & Gooch, C. Covered manure storage cost calculator. http://northeast.manuremanagement.cornell.edu/Pages/Assessment_Tools/Covered_Storage_Calculator.html (College of Agricultural Life Sciences, Cornell University, 2008).

[9] Knighton J, Pluer EM, Prestigiacomo AR, Effler SW, Walter MT (2017). Topographic wetness guided dairy manure applications to reduce stream nutrient loads in Central New York, USA. J. Hydrol. Reg..

[10] Sharara M (2017). Spatially explicit methodology for coordinated manure management in shared watersheds. J. Environ. Manage..

[11] Ribaudo MO, Gollehon NR, Agapoff J (2003). Land application of manure by animal feeding operations: Is more land needed?. J. Soil Water Conserv..

[12] Kassem N, Sills D, Posmanik R, Blair C, Tester JW (2020). Combining anaerobic digestion and hydrothermal liquefaction in the conversion of dairy waste into energy: A techno economic model for New York state. Waste Manage..

[13] Jahne MA, Rogers SW, Holsen TM, Grimberg SJ, Ramler IP (2015). Emission and dispersion of bioaerosols from dairy manure application sites: Human health risk assessment. Environ. Sci. Technol..

[14] Ghafoori E, Flynn PC, Feddes JJ (2007). Pipeline vs truck transport of beef cattle manure. Biomass Bioenergy.

[15] Font-Palma C (2018). Methods for the treatment of cattle manure—A review. C J. Carbon Res..

[16] Zhang RH, Westerman PW (1997). Solid-liquid separation of annual manure for odor control and nutrient management. Appl. Eng. Agric..

[17] Møller HB, Lund I, Sommer SG (2000). Solid–liquid separation of livestock slurry: Efficiency and cost. Bioresour. Technol..

[18] Burton CH (2007). The potential contribution of separation technologies to the management of livestock manure. Livest. Sci..

[19] Welsh R, Grimberg S, Gillespie GW, Swindal M (2010). Technoscience, anaerobic digester technology and the dairy industry: Factors influencing North Country New York dairy farmer views on alternative energy technology. Renew. Agric. Food Syst..

[20] Leytem AB, Dungan RS, Bjorneberg DL, Koehn AC (2011). Emissions of ammonia, methane, carbon dioxide, and nitrous oxide from dairy cattle housing and manure management systems. J. Environ. Qual..

[21] VanderZaag A (2017). Potential methane emission reductions for two manure treatment technologies. Environ. Technol..

[22] Lehmann J, Joseph S (2015). Biochar for Environmental Management: Science, Technology and Implementation.

[23] Enders A, Hanley K, Whitman T, Joseph S, Lehmann J (2012). Characterization of biochars to evaluate recalcitrance and agronomic performance. Bioresour. Technol..

[24] Ahmad M (2014). Biochar as a sorbent for contaminant management in soil and water: A review. Chemosphere.

[25] Hollister CC, Bisogni JJ, Lehmann J (2013). Ammonium, nitrate, and phosphate sorption to and solute leaching from biochars prepared from corn stover (*Zea mays* L.) and oak wood (*Quercus* spp.). J. Environ. Qual..

[26] Mohan D, Sarswat A, Ok YS, Pittman CU (2014). Organic and inorganic contaminants removal from water with biochar, a renewable, low cost and sustainable adsorbent—A critical review. Bioresour. Technol..

[27] Wang B, Lehmann J, Hanley K, Hestrin R, Enders A (2016). Ammonium retention by oxidized biochars produced at different pyrolysis temperatures and residence times. RSC Adv..

[28] Shaaban M (2018). A concise review of biochar application to agricultural soils to improve soil conditions and fight pollution. J. Environ. Manage..

[29] Hestrin R (2019). Fire-derived organic matter retains ammonia through covalent bond formation. Nat. Commun..

[30] Garner W (1973). The Disposal of Cattle Feedlot Wastes by Pyrolysis.

[31] Schouten S, van Groenigen JW, Oenema O, Cayuela ML (2012). Bioenergy from cattle manure? Implications of anaerobic digestion and subsequent pyrolysis for carbon and nitrogen dynamics in soil. Glob. Change Biol. Bioenerg..

[32] Novak JM, Johnson MG, Ok YS, Tsang DCW, Bolan N, Novak JM (2019). Elemental and spectroscopic characterization of low-temperature (350 °C) lignocellulosic-and manure-based designer biochars and their use as soil amendments. Biochar from Biomass and Waste: Fundamentals and Applications.

[33] Zhao N, Lehmann J, You F (2020). Poultry waste valorization via pyrolysis technologies: Economic and environmental life cycle optimization for sustainable bioenergy systems. ACS Sustain. Chem. Eng..

[34] Struhs E, Mirkouei A, You Y, Mohajeri A (2020). Techno-economic and environmental assessments for nutrient-rich biochar production from cattle manure: A case study in Idaho, USA. Appl. Energy.

[35] Ro KS, Cantrell KB, Hunt PG (2010). High-temperature pyrolysis of blended animal manures for producing renewable energy and value-added biochar. Ind. Eng. Chem. Res..

[36] Cely P, Gascó G, Paz-Ferreiro J, Méndez A (2015). Agronomic properties of biochars from different manure wastes. J. Anal. Appl. Pyrolysis.

[37] Wang T, Arbestain MC, Hedley M, Bishop P (2012). Chemical and bioassay characterisation of nitrogen availability in biochar produced from dairy manure and biosolids. Organ. Geochem..

[38] Cantrell KB (2012). Impact of pyrolysis temperature and manure source on physicochemical characteristics of biochar. Bioresour. Technol..

[39] Krounbi L (2019). Biological and thermochemical conversion of human solid waste to soil amendments. Waste Manage..

[40] Figueiredo CCD (2020). Sewage sludge biochar increases nitrogen fertilizer recovery: Evidence from a 15 N tracer field study. Soil Use Manage..

[41] Krounbi L (2020). Sequential ammonia and carbon dioxide adsorption on pyrolyzed biomass to recover waste stream nutrients. ACS Sustain. Chem. Eng..

[42] Fernandez-Lopez M (2015). Life cycle assessment of swine and dairy manure: pyrolysis and combustion processes. Bioresour. Technol..

[43] Taghizadeh-Toosi A, Clough TJ, Sherlock RR, Condron LM (2012). Biochar adsorbed ammonia is bioavailable. Plant Soil.

[44] Van Humbeck JF (2014). Ammonia capture in porous organic polymers densely functionalized with Brønsted acid groups. J. Am. Chem. Soc..

[45] Shen W, Fan W (2013). Nitrogen-containing porous carbons: Synthesis and application. J. Mat. Chem. A..

[46] Shafeeyan MS, Daud WMAW, Houshmand A, Arami-Niya A (2011). Ammonia modification of activated carbon to enhance carbon dioxide adsorption: Effect of pre-oxidation. Appl. Surf. Sci..

[47] Taghizadeh-Toosi A, Clough TJ, Sherlock RR, Condron LM (2012). A wood based low-temperature biochar captures NH_3_-N generated from ruminant urine-N, retaining its bioavailability. Plant Soil.

[48] Environmental Protection Agency (1995). A Guide to the Biosolids Risk Assessments for the EPA Part 503 Rule.

[49] Cárdenas-Aguiar E, Gascó G, Paz-Ferreiro J, Méndez A (2019). Thermogravimetric analysis and carbon stability of chars produced from slow pyrolysis and hydrothermal carbonization of manure waste. J. Anal. Appl. Pyrolysis.

[50] Pettygrove GS (2003). Mineralization of nitrogen in dairy manure water. Western Nutr. Manag. Conf..

[51] Grunert O (2016). Growing media constituents determine the microbial nitrogen conversions in organic growing media for horticulture. Microb. Biotechnol..

[52] Yeh JT, Resnik KP, Rygle K, Pennline HW (2005). Semi-batch absorption and regeneration studies for CO_2_ capture by aqueous ammonia. Fuel Process Technol..

[53] Dutcher B, Fan M, Russell AG (2015). Amine-based CO_2_ capture technology development from the beginning of 2013: A review. ACS Appl. Mat. Interfaces.

[54] McLeod A, Jefferson B, McAdam EJ (2014). Biogas upgrading by chemical absorption using ammonia rich absorbents derived from wastewater. Water Res..

[55] NY State Comptroller. A profile of agriculture in New York State. https://www.osc.state.ny.us/files/reports/special-topics/pdf/agriculture-report-2019.pdf (Office of Budget and Policy Analysis, Office of the New York State Comptroller, 2019).

[56] Nennich T, Burns RT (2013). Development of standard methods to estimate manure production and nutrient characteristics from dairy cattle. IX 1-Animal, Agricultural and Food Processing Wastes.

[57] Cornell University. Fertilizers for corn. https://fieldcrops.cals.cornell.edu/corn/fertilizers-corn/ (College of Agricultural and Life Sciences (CALS), 2021).

[58] R Computing Team (2000). R Language Definition.

[59] Wilkinson L (2011). ggplot2: Elegant graphics for data analysis by Wickham, H. Biometrics.

[60] Lenth RV (2016). Least-squares means: The R package lsmeans. J. Stat. Softw..

[61] Graves, S., Piepho, H. P., Selzer, L. & Dorai-Raj, S. multcompView: Visualizations of paired comparisons. R package version 0.1-5; 46. https://CRAN.R-project.org/package=multcompView (2012).

