# Supplementary material for: Plant uptake of nitrogen adsorbed to biochars made from dairy manure
Source: Sci Rep. 2021 Jul 22;11:15001. doi: 10.1038/s41598-021-94337-8 (PMC8298528; doi:10.1038/s41598-021-94337-8)
Supplement: Supplementary file 1 — Supplementary Information. [file 41598_2021_94337_MOESM1_ESM.docx]

**Supplementary Methods**

**Plant uptake of nitrogen adsorbed to biochars made from dairy manure**

Leilah Krounbi^1^, Akio Enders^1,2^, John Gaunt^2^, Margaret Ball^2^ & Johannes Lehmann^1,3*^

*^1^Soil and Crop Sciences, School of Integrative Plant Science, College of Agriculture and Life Sciences, Cornell University, Ithaca, NY 14853, USA*

*^2^bio365 LLC, Ithaca, NY, 14850*

*^3^Cornell Atkinson Center for Sustainability, Cornell University, Ithaca, NY 14853, USA*

*corresponding author, Email: [CL273@cornell.edu](mailto:CL273@cornell.edu)

Table SI 1 Composition of nutrient powder mixture used for the growth trial.

| Reagent | Amount in mixture (g kg^-1^) |
| --- | --- |
| CaHPO_4_^2-^·H_2_O | 61.47 |
| K_2_CO_3_ | 120.24 |
| MgO | 54.0 |
| ZnSO_4_·7H_2_O | 2.48 |
| NaCl | 54.15 |
| CaCO_3_ | 688.46 |
| K_2_SO_4_ | 18.95 |

Table SI 2 Description of growth-trial amendment treatments and irrigation amounts. All amendments were added to 41.77 g of TH6 peat potting media and mixed thoroughly while dry. The bulk densities used for calculations are: (1) TH6 peat potting media = 0.14 g cm^-3^, (2) manure biochar = 0.32 g cm^-3^, (3) wood biochar = 0.13 g cm^-3^.

| Amend. | N-form | Urea and biochar | Biochar (ton ha^-1^) | Nutrient powder  (ton ha^-1^) | Urea-N  (kg ha^-1^) | Urea  (kg ha^-1^) | Initial irrigation (m^3^ ha^-1^) |
| --- | --- | --- | --- | --- | --- | --- | --- |
| None | None | 0.00 | 0.00 | 2.08 | 0.00 | 0.00 | 503.36 |
| 0.25x fert | Urea | 0.25-fold of wood biochar N uptake (Table 1) | 0.00 | 2.08 | 29.46 | 63.15 | 503.36 |
| 0.5x fert | Urea | 0.5-fold of wood biochar N uptake | 0.00 | 2.08 | 58.89 | 126.31 | 503.36 |
| 1x fert | Urea | 1-fold (same amount) of wood biochar N uptake | 0.00 | 2.08 | 117.79 | 252.65 | 503.36 |
| 1.5x fert | Urea | 1.5-fold of wood biochar N uptake | 0.00 | 2.08 | 176.68 | 378.96 | 503.36 |
| Manure biochar NH_3_+CO_2_ | NH_3_ gas | Equivalent on C basis to wood biochar (Table 1) | 24.16 | 0.00 | 0.00 | 0.00 | 516.78 |
| Manure biochar+ slurry | Slurry | Equivalent on C basis to wood biochar | 24.16 | 0.00 | 0.00 | 0.00 | 516.78 |
| Manure biochar+1x fertilizer | Urea | Equivalent on C basis to wood biochar + urea-N 1-fold of wood biochar N uptake | 24.16 | 0.00 | 117.79 | 252.65 | 516.78 |
| Manure biochar | None | Equivalent on C basis to wood biochar | 24.16 | 0.00 | 0.00 | 0.00 | 516.78 |
| Wood biochar NH_3_+CO_2_ | NH3 gas | 10% of pot volume | 12.08 | 2.08 | 0.00 | 0.00 | 577.18 |
| Wood biochar+ slurry | Slurry | 10% of pot volume | 12.08 | 2.08 | 0.00 | 0.00 | 577.18 |
| Wood biochar+1x fertilizer | Urea | 10% of pot volume + urea-N 1-fold of wood biochar N uptake | 12.08 | 2.08 | 117.79 | 252.65 | 577.18 |
| Wood biochar | None | 10% of pot volume | 12.08 | 2.08 | 0.00 | 0.00 | 577.18 |

Table SI 3 Description of urea-N additions (1x) in greenhouse trial based on six replicate measurements of N uptake of wood biochar after exposure to CO_2_+NH_3_.

| Cycle of gas adsorption | Description | N  (% w w^-1^) | ΔN wood biochar [CO_2_+NH_3_ - wood biochar] (% w w^-1^) | Urea-N added to 3.6 g wood biochar [1x] (mg) | Urea-N addition in 1x treatment (g kg^-1^) |
| --- | --- | --- | --- | --- | --- |
| 1 | Wood biochar+NH_3_ | 2.16 | 0.98 | 35.10 | 4.90 |
| 2 | Wood biochar+NH_3_+CO_2_ | 1.48 |  |  |  |
| 3 | Wood biochar+NH_3_+CO_2_+NH_3_ | 1.65 |  |  |  |
| 4 | Wood biochar+NH_3_+CO_2_+NH_3_+CO_2_ | 0.67 |  |  |  |
| 5 | Wood biochar+NH_3_+CO_2_+NH_3_+CO_2_+NH_3_ | 1.48 |  |  |  |
| 6 | Wood biochar+NH_3_+CO_2_+NH_3_+CO_2_+NH_3_+CO_2_ | 1.41 |  |  |  |
|  | Average n = 6 | 1.48 |  |  |  |
|  | Unexposed wood biochar (control) average n = 5 | 0.50 |  |  |  |

Table SI 4 Germination, mass of dry shoot and root biomass, and total carbon and nitrogen in shoot and root biomass of marigold, radish, and tomato plants. Letters not in brackets indicate significant differences between amendments within plant and biomass type while letters in brackets indicate significant differences between amendments and plant type within each biomass type (p < 0.05; n = 4).

| Plant | Amendment | Germination | Shoot biomass (dry) | Shoot nitrogen | Shoot carbon | Root biomass (dry) | Root nitrogen | Root carbon |
| --- | --- | --- | --- | --- | --- | --- | --- | --- |
|  |  | (%) | (g pot^-1^) | (% w w^-1^) | (% w w^-1^) | (g pot^-1^) | (% w w^-1^) | (% w w^-1^) |
| Marigold | 0x fert | 80.04 ± 23.07 a | 1.99 ± 0.26 ab | 1.42 ± 0.38 ab | 44.56 ± 1.04 a | 0.82 ± 0.14 ab | 0.99 ± 0.15 a | 43.53 ± 0.78 a |
| Marigold | 0.25x fert | 55.03 ± 41.23 a | 2.38 ± 0.36 ab | 1.30 ± 0.24 ab | 44.64 ± 1.26 a | 1.01 ± 0.18 ab | 0.94 ± 0.08 a | 43.00 ± 1.01 a |
| Marigold | 0.5x fert | 75.01 ± 34.20 a | 2.62 ± 0.41 a | 1.14 ± 0.17 ab | 45.32 ± 0.90 a | 0.97 ± 0.17 ab | 0.95 ± 0.11 a | 43.90 ± 0.59 a |
| Marigold | 1x fert | 55.02 ± 34.22 a | 2.65 ± 0.51 a | 1.62 ± 0.51 ab | 45.2 ± 1.02 a | 0.98 ± 0.13 ab | 1.07 ± 0.11 a | 44.77 ± 0.48 a |
| Marigold | 1.5x fert | 55.04 ± 19.20 a | 2.80 ± 0.39 a | 1.86 ± 0.16 a | 45.22 ± 0.31 a | 0.86 ± 0.08 ab | 1.10 ± 0.14 a | 43.01 ± 3.31 a |
| Marigold | Manure biochar | 80.01 ± 16.31 a | 1.93 ± 0.13 ab | 1.27 ± 0.26 ab | 44.45 ± 0.18 a | 0.76 ± 0.26 abc | 1.00 ± 0.27 a | 42.41 ± 0.80 a |
| Marigold | Manure biochar+ slurry | 60.00 ± 43.21 a | 1.96 ± 0.42 ab | 1.39 ± 0.61 ab | 44.01 ± 1.14 a | 0.97 ± 0.14 ab | 0.89 ± 0.08 a | 42.81 ± 0.90 a |
| Marigold | Manure biochar CO_2_+NH_3_ | 65.02 ± 47.30 a | 1.24 ± 0.83 bc | 1.59 ± 1.16 ab | 33.47 ± 22.4 ab | 0.59 ± 0.41 bc | 0.86 ± 0.62 ab | 31.93 ± 21.34 a |
| Marigold | Dairy manure biochar+1x | 30.04 ± 20.03 a | 0.60 ± 1.19 c | 0.47 ± 0.94 b | 11.42 ± 22.90 b | 0.20 ± 0.41 c | 0.22 ± 0.44 b | 9.04 ± 18.08 b |
| Marigold | Wood biochar | 65.02 ± 19.17 a | 2.62 ± 0.13 a | 0.94 ± 0.12 ab | 45.09 ± 0.58 a | 0.94 ± 0.25 ab | 0.88 ± 0.29 a | 42.51 ± 2.36 a |
| Marigold | Wood biochar+ slurry | 35.01 ± 34.22 a | 2.33 ± 0.29 ab | 1.09 ± 0.08 ab | 44.61 ± 0.54 a | 0.81 ± 0.15 ab | 0.96 ± 0.19 a | 43.83 ± 0.50 a |
| Marigold | Wood biochar NH_3_+CO_2_ | 60.04 ± 43.20 a | 2.70 ± 0.33 a | 1.39 ± 0.28 ab | 45.80 ± 0.41 a | 0.96 ± 0.14 ab | 0.94 ± 0.16 a | 43.72 ± 0.64 a |
| Marigold | Wood biochar+1x | 60.04 ± 28.34 a | 3.10 ± 0.17 a | 1.37 ± 0.05 ab | 46.01 ± 0.22 a | 1.20 ± 0.17 a | 0.91 ± 0.10 a | 43.03 ± 2.53 a |
| Radish | 0x fert | 90.01 ± 11.63 a | 0.77 ± 0.14 b | 1.43 ± 0.40 abc | 41.91 ± 1.22 ab | 2.31 ± 0.27 c | 0.81 ± 0.17 a | 40.94 ± 4.18 a |
| Radish | 0.25x fert | 70.00 ± 11.60 a | 1.05 ± 0.31 ab | 1.80 ± 0.33 abc | 42.90 ± 0.71 a | 2.81 ± 0.47 bc | 0.78 ± 0.25 a | 41.12 ± 2.88 a |
| Radish | 0.5x fert | 80.04 ± 16.33 a | 0.89 ± 0.12 ab | 1.86 ± 0.55 abc | 42.22 ± 1.22 ab | 3.20 ± 0.29 abc | 0.83 ± 0.15 a | 41.71 ± 2.34 a |
| Radish | 1x fert | 65.04 ± 34.23 a | 1.08 ± 0.17 ab | 1.96 ± 0.28 abc | 41.91 ± 2.12 ab | 3.05 ± 0.61 abc | 1.07 ± 0.26 a | 43.11 ± 4.46 a |
| Radish | 1.5x fert | 85.00 ± 19.24 a | 1.34 ± 0.34 a | 2.05 ± 0.68 ab | 43.40 ± 1.45 a | 3.04 ± 0.90 abc | 1.00 ± 0.26 a | 39.61 ± 7.00 a |
| Radish | Manure biochar | 90.02 ± 20.01 a | 0.74 ± 0.15 b | 1.34 ± 0.34 abc | 40.38 ± 1.91 ab | 2.91 ± 0.73 abc | 0.72 ± 0.06 a | 42.80 ± 1.52 a |
| Radish | Manure biochar+ slurry | 90.01 ± 20.00 a | 1.00 ± 0.13 ab | 1.19 ± 0.29 bc | 39.14 ± 3.50 b | 2.92 ± 0.32 abc | 0.68 ± 0.03 a | 40.52 ± 4.31 a |
| Radish | Manure biochar CO_2_+NH_3_ | 65.01 ± 10.02 a | 0.98 ± 0.21 ab | 2.12 ± 0.46 a | 41.80 ± 1.31 ab | 3.50 ± 0.36 abc | 1.01 ± 0.22 a | 40.61 ± 2.55 a |
| Radish | Manure biochar+1x | 75.01 ± 19.20 a | 0.94 ± 0.12 ab | 1.51 ± 0.33 abc | 41.11 ± 0.41 ab | 4.10 ± 0.20 a | 0.80 ± 0.06 a | 42.69 ± 0.82 a |
| Radish | Wood biochar | 90.04 ± 20.03 a | 0.84 ± 0.12 ab | 1.10 ± 0.12 c | 42.75 ± 0.90 ab | 3.13 ± 0.27 abc | 0.72 ± 0.15 a | 42.56 ± 1.54 a |
| Radish | Wood biochar+ slurry | 90.04 ± 11.59 a | 0.91 ± 0.26 ab | 1.17 ± 0.20 bc | 42.28 ± 0.47 ab | 2.82 ± 0.24 bc | 0.78 ± 0.07 a | 42.33 ± 1.32 a |
| Radish | Wood biochar NH_3_+CO_2_ | 80.01 ± 16.32 a | 1.23 ± 0.26 ab | 1.52 ± 0.19 abc | 42.91 ± 0.71 a | 3.81 ± 0.44 ab | 0.83 ± 0.11 a | 42.84 ± 1.01 a |
| Radish | Wood biochar+1x | 90.03 ± 11.55 a | 1.15 ± 0.11 ab | 1.39 ± 0.19 abc | 42.3 ± 1.11 ab | 3.88 ± 0.61 ab | 0.84 ± 0.08 a | 43.61 ± 0.30 a |
| Tomato | 0x fert | 85.03 ± 19.21 a | 3.04 ± 0.37 bcd | 1.13 ± 0.12 c | 42.11 ± 0.54 abcd | 0.47 ± 0.09 bc | 1.18 ± 0.08 c | 42.44 ± 0.52 abcde |
| Tomato | 0.25x fert | 85.02 ± 19.22 a | 3.37 ± 0.57 abcd | 1.19 ± 0.17 bc | 42.3 ± 0.70 abcd | 0.56 ± 0.10 abc | 1.26 ± 0.11 bc | 42.82 ± 0.31 ab |
| Tomato | 0.5x fert | 55.04 ± 37.90 a | 3.65 ± 0.27 abcd | 1.27 ± 0.15 bc | 42.6 ± 0.47 abcd | 0.55 ± 0.13 bc | 1.24 ± 0.16 bc | 43.33 ± 0.60 a |
| Tomato | 1x fert | 65.01 ± 44.36 a | 3.31 ± 0.67 abcd | 1.68 ± 0.52 c | 42.82 ± 0.72 abc | 0.52 ± 0.11 bc | 1.53 ± 0.30 ab | 43.60 ± 0.43 a |
| Tomato | 1.5x fert | 100.21 ± 0.02 a | 2.77 ± 0.51 d | 2.41 ± 0.38 a | 43.3 ± 0.71 ab | 0.34 ± 0.13 c | 1.76 ± 0.14 a | 42.60 ± 0.65 abc |
| Tomato | Manure biochar | 85.0 ± 19.15 a | 2.84 ± 0.26 cd | 1.01 ± 0.11 c | 41.2 ± 0.32 d | 0.52 ± 0.05 bc | 1.09 ± 0.06 c | 41.24 ± 0.42 e |
| Tomato | Manure biochar+ slurry | 85.04 ± 30.02 a | 3.06 ± 0.36 bcd | 1.00 ± 0.13 c | 41.1 ± 0.47 d | 0.56 ± 0.09 abc | 1.21 ± 0.11 c | 41.27 ± 0.54 de |
| Tomato | Manure biochar CO_2_+NH_3_ | 70.01 ± 25.78 a | 3.99 ± 0.21 ab | 1.12 ± 0.12 c | 41.6 ± 0.17 cd | 0.56 ± 0.03 bc | 1.29 ± 0.07 bc | 42.43 ± 0.87 abcde |
| Tomato | Manure biochar+1x | 55.02 ± 37.94 a | 3.77 ± 0.38 abc | 1.14 ± 0.17 c | 42.21 ± 0.60 abcd | 0.63 ± 0.11 ab | 1.13 ± 0.07 c | 41.51 ± 0.80 cde |
| Tomato | Wood biochar | 100.24 ± 0.00 a | 3.09 ± 0.23 abcd | 0.96 ± 0.06 c | 42.01 ± 0.41 abcd | 0.59 ± 0.08 ab | 1.02 ± 0.07 c | 42.52 ± 0.40 abcd |
| Tomato | Wood biochar+ slurry | 90.01 ± 11.62 a | 3.21 ± 0.34 abcd | 1.07 ± 0.12 c | 41.81 ± 0.92 bcd | 0.56 ± 0.16 bc | 1.09 ± 0.06 c | 41.80 ± 0.34 bcde |
| Tomato | Wood biochar NH_3_+CO_2_ | 90.01 ± 11.62 a | 3.69 ± 0.26 abcd | 1.21 ± 0.10 bc | 43.38 ± 0.80 a | 0.64 ± 0.07 ab | 1.14 ± 0.04 c | 43.11 ± 0.21 a |
| Tomato | Wood biochar+1x | 85.00 ± 10.04 a | 4.06 ± 0.42 a | 1.18 ± 0.06 bc | 43.54 ± 0.80 a | 0.80 ± 0.03 a | 1.18 ± 0.07 c | 43.17 ± 0.12 a |

**
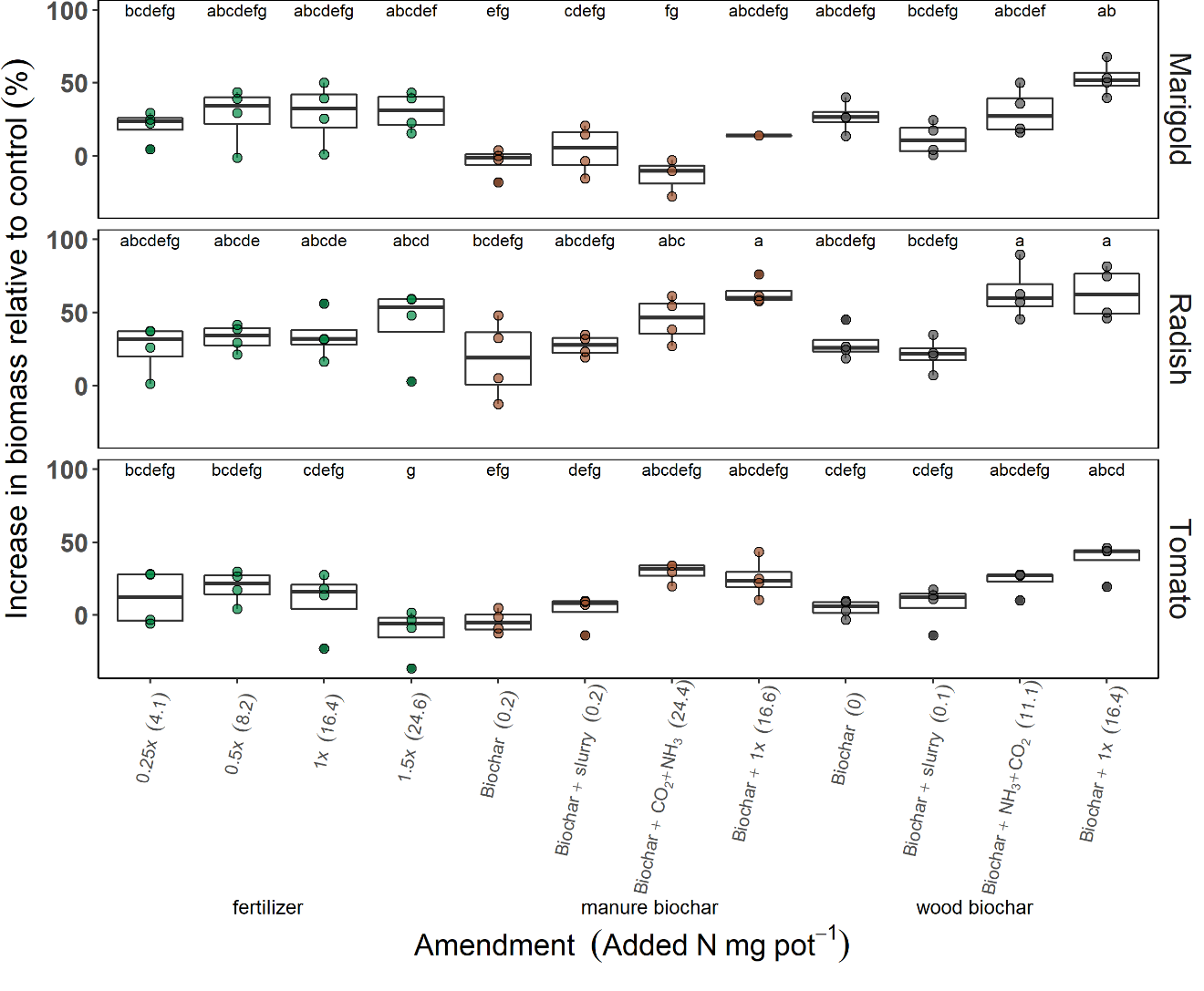
**

Figure SI 1. Increase in plant biomass (sum of root and shoot biomass) grown with urea fertilizer (green points), manure biochar (brown points) or wood biochar (gray points) amendments relative to unamended plants (0x). The amount of plant-available N in each type of amendment is listed in parentheses. Letters above the bars indicate significant differences between amendments and plant type as calculated from a two-way anova (*p* < 0.05; n = 4; whiskers indicate standard errors).

**
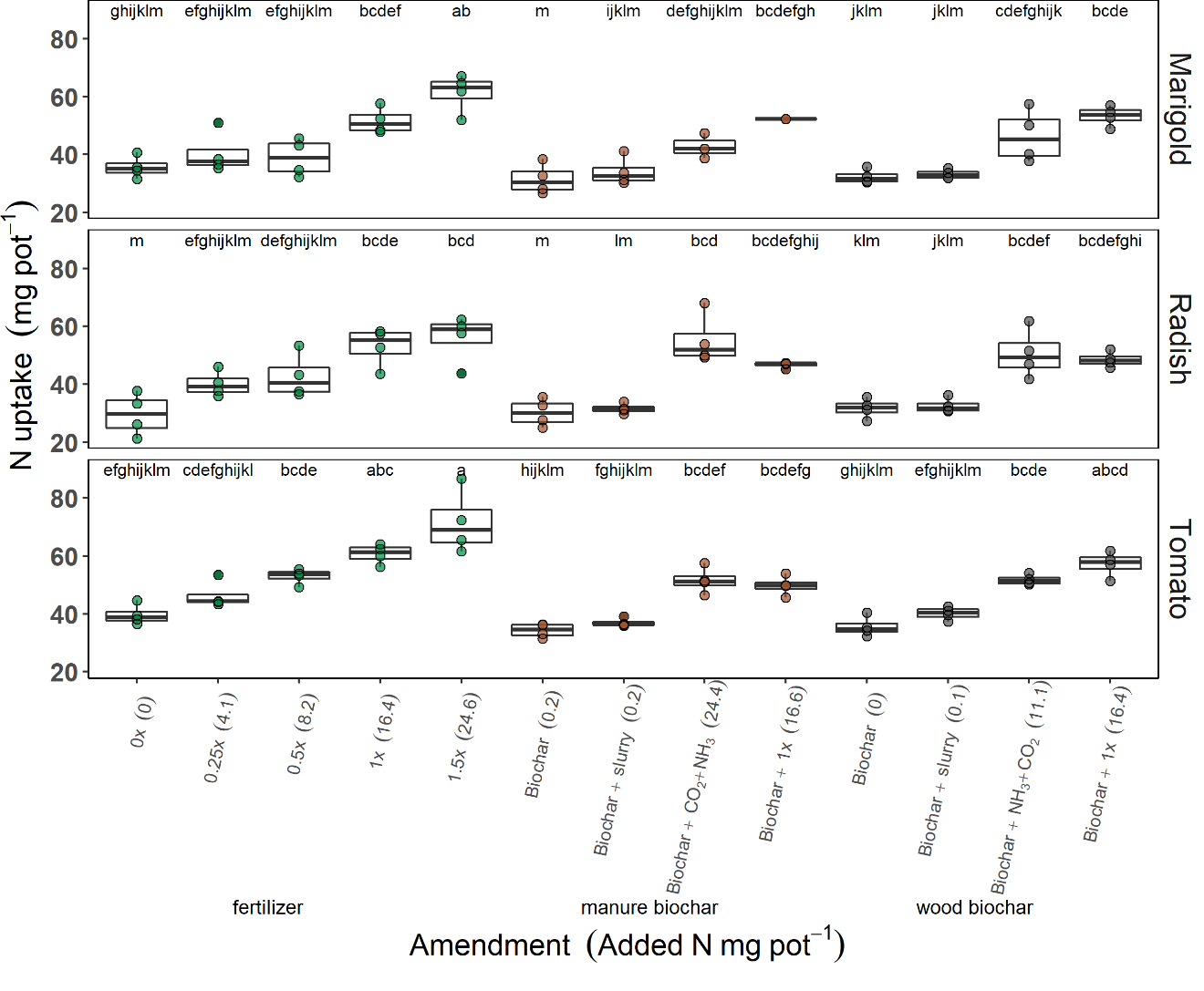
**

Figure SI 2. Total nitrogen uptake in shoot and root biomass of plants grown with urea fertilizer (green dots), manure biochar (brown dots), or wood biochar (grey dots) amendments or no amendments (0x). Letters above the bars indicate significant differences between amendments and plant type as calculated from a two-way anova (*p* < 0.05; n = 4).

Table SI 5 The average ± standard deviation of the pH of potting mix and amendments after 40 days, total plant biomass (shoot and root combined) and relative increase in plant biomass relative to unamended plants (0x), total N plant uptake (shoot and root combined), and relative increase in biomass N uptake in comparison to unamended plants. Letters not in brackets indicate significant differences from a one-way anova between amendments within plant type while letters within square brackets indicate significant differences from a two-way anova between amendments and plant type (p < 0.05; n = 4).

| Plant | Amendment | pH after 40 days | Plant N uptake (mg pot^-1^) | Relative increase in plant N uptake  (% w w^-1^) | Plant biomass  (g pot^-1^) | Relative increase in plant biomass  (% w w^-1^) |
| --- | --- | --- | --- | --- | --- | --- |
| Marigold | 0x fert | 6.35 ± 0.13 cd [ghij] | 35.63 ± 3.87 de [ghijklm] | NA | 2.81 ± 0.36 bc [hij] | NA |
| Marigold | 0.25x fert | 6.28 ± 0.14 cd [hij] | 40.37 ± 7.21 bcde [efghijklm] | 13.28 ± 20.25 bcde [ghijklm] | 3.38 ± 0.31 abc [defghij] | 20.27 ± 10.89 abc [bcdefg] |
| Marigold | 0.5x fert | 6.30 ± 0.04 cd [hij] | 38.97 ± 6.45 cde [efghijklm] | 9.36 ± 18.10 cde  [ghijklm] | 3.59 ± 0.57 ab [cdefghij] | 27.73 ± 20.15 ab [abcdefg] |
| Marigold | 1x fert | 6.15 ± 0.14 d [j] | 51.63 ± 4.49 abc [bcdef] | 44.89 ± 12.61 abc  [bcdefghi] | 3.63 ± 0.60 ab [cdefghij] | 28.98 ± 21.20 ab [abcdefg] |
| Marigold | 1.5x fert | 6.25 ± 0.11 cd [hij] | 61.36 ± 6.64 a [ab] | 72.20 ± 18.65 a  [abcd] | 3.66 ± 0.37 ab [cdefghij] | 30.22 ± 13.25 ab [abcdef] |
| Marigold | Manure biochar | 7.46 ± 0.16 a [a] | 31.50 ± 5.24 e [m] | -11.60 ± 14.71 e  [lm] | 2.69 ± 0.27 bc [ij] | -4.27 ± 9.67 bc [efg] |
| Marigold | Manure biochar+slurry | 7.35 ± 0.43 a [ab] | 34.10 ± 4.93 de [ijklm] | -4.30 ± 13.83 de  [jklm] | 2.93 ± 0.46 bc [ghij] | 4.00 ± 16.51 bc [cdefg] |
| Marigold | Manure biochar CO_2_+NH_3_ | 7.41 ± 0.24 a [abc] | 42.74 ± 4.38 bcde [defghijklm] | 19.95 ± 12.29 bcde [efghijklm] | 2.43 ± 0.36 c [j] | -13.72 ± 12.77 c [fg] |
| Marigold | Manure biochar+1x | 7.35 ± 0.04 a [abc] | 52.28 ± 0.00 abc [bcdefgh] | 46.71 ± 0.00 abc [abcdefghij] | 3.20 ± 0.00 abc [defghij] | 13.78 ± 0.00 abc [abcdefg] |
| Marigold | Wood biochar | 6.53 ± 0.19 cd [defghij] | 32.40 ± 2.46 e [jklm] | -9.08 ± 6.92 e [klm] | 3.56 ± 0.31 ab [defghij] | 26.58 ± 10.89 ab [abcdefg] |
| Marigold | Wood biochar+slurry | 7.03 ± 0.18 ab [abcdef] | 33.20 ± 1.63 de [jklm] | -6.84 ± 4.56 de [jklm] | 3.14 ± 0.31 bc [efghij] | 11.64 ± 11.16 bc [bcdefg] |
| Marigold | Wood biochar NH_3_+CO_2_ | 6.62 ± 0.13 bc [defghij] | 46.44 ± 9.15 bcd [cdefghijk] | 30.33 ± 25.67 bcd [defghijkl] | 3.66 ± 0.44 ab [cdefghij] | 30.22 ± 15.82 ab [abcdef] |
| Marigold | Wood biochar+1x | 6.51 ± 0.15 cd [defghij] | 53.33 ± 3.45 ab [bcde] | 49.66 ± 9.67 ab [abcdefgh] | 4.30 ± 0.33 a [abcdef] | 52.80 ± 11.58 a [ab] |
| Radish | 0x fert | 6.48 ± 0.12 cd [efghij] | 29.60 ± 7.33 d [m] | NA | 3.08 ± 0.27 c [fghij] | NA |
| Radish | 0.25x fert | 6.51 ± 0.16 cd [defghij] | 40.06 ± 4.41 bcd [efghijklm] | 35.33 ± 14.89 bcd [cdefghij] | 3.87 ± 0.52 abc [abcdefghi] | 25.39 ± 16.91 ab [abcdefg] |
| Radish | 0.5x fert | 6.34 ± 0.14 d [ghij] | 42.74 ± 7.71 abcd [defghijklm] | 44.37 ± 26.04 abcd [bcdefghi] | 4.09 ± 0.28 abc [abcdefgh] | 32.69 ± 9.13 ab [abcde] |
| Radish | 1x fert | 6.35 ± 0.15 d [ghij] | 53.04 ± 6.73 ab [bcde] | 79.17 ± 22.74 ab [abc] | 4.13 ± 0.50 abc [abcdefg] | 34.06 ± 16.35 ab [abcde] |
| Radish | 1.5x fert | 6.37 ± 0.14 d [ghij] | 55.94 ± 8.38 a [bcd] | 88.96 ± 28.30 a [a] | 4.39 ± 0.83 ab [abcde] | 42.25 ± 26.80 ab [abcd] |
| Radish | Manure biochar | 7.22 ± 0.10 ab [abc] | 30.19 ± 4.82 d [m] | 1.99 ± 16.27 d [ijklm] | 3.65 ± 0.84 bc [cdefghij] | 18.25 ± 27.22 b [bcdefg] |
| Radish | Manure biochar+slurry | 7.31 ± 0.12 a [abc] | 31.60 ± 1.78 d [lm] | 6.74 ± 6.00 d [hijklm] | 3.93 ± 0.22 abc [abcdefghi] | 27.33 ± 7.19 ab [abcdefg] |
| Radish | Manure biochar CO_2_+NH_3_ | 7.25 ± 0.13 a [abc] | 55.28 ± 8.68 a [bcd] | 86.75 ± 29.34 a [ab] | 4.48 ± 0.48 ab [abcd] | 45.26 ± 15.45 ab [abc] |
| Radish | Manure biochar+1x | 7.02 ± 0.56 ab [abcdef] | 46.72 ± 1.03 abc [bcdefghij] | 57.82 ± 3.49 abc [abcdef] | 5.04 ± 0.27 a [a] | 63.42 ± 8.62 a [a] |
| Radish | Wood biochar | 6.74 ± 0.04 bcd [cdefghi] | 31.68 ± 3.45 d [klm] | 7.01 ± 11.64 d [hijklm] | 3.97 ± 0.35 abc [abcdefghi] | 28.79 ± 11.34 ab [abcdefg] |
| Radish | Wood biochar+slurry | 6.90 ± 0.16 abc [abcdefg] | 32.58 ± 2.55 cd [jklm] | 10.05 ± 8.61 cd [ghijklm] | 3.74 ± 0.35 bc [bcdefghij] | 21.17 ± 11.28 b [bcdefg] |
| Radish | Wood biochar NH_3_+CO_2_ | 6.45 ± 0.06 cd [fghij] | 50.52 ± 8.56 ab [bcdef] | 70.65 ± 28.92 ab [abcd] | 5.05 ± 0.58 a [a] | 63.67 ± 18.83 a [a] |
| Radish | Wood biochar+1x | 6.48 ± 0.14 cd [efghij] | 48.51 ± 2.66 ab [bcdefghi] | 63.88 ± 8.97 ab [abcde] | 5.03 ± 0.55 a [ab] | 63.10 ± 17.80 a [a] |
| Tomato | 0x fert | 6.11 ± 0.07 d [j] | 39.70 ± 3.52 def [efghijklm] | NA | 3.52 ± 0.43 bcd [defghij] | NA |
| Tomato | 0.25x fert | 6.15 ± 0.18 d [j] | 46.36 ± 4.73 cde [cdefghijkl] | 16.79 ± 11.92 cde [fghijklm] | 3.93 ± 0.66 abcd [abcdefghi] | 11.81 ± 18.76 abcd [bcdefg] |
| Tomato | 0.5x fert | 6.33 ± 0.55 cd [ghij] | 52.98 ± 2.68 bc [bcde] | 33.46 ± 6.74 bc [defghijk] | 4.20 ± 0.40 abcd [abcdefg] | 19.49 ± 11.37 abcd [bcdefg] |
| Tomato | 1x fert | 6.17 ± 0.02 d [ij] | 60.72 ± 3.40 ab [abc] | 52.95 ± 8.58 ab [abcdefg] | 3.84 ± 0.78 abcd [abcdefghi] | 9.10 ± 22.11 abcd [cdefg] |
| Tomato | 1.5x fert | 6.36 ± 0.30 cd [ghij] | 71.55 ± 11.10 a [a] | 80.23 ± 27.97 a [ab] | 3.11 ± 0.60 d [efghij] | -11.59 ± 16.98 d [g] |
| Tomato | Manure biochar | 7.08 ± 0.12 a [abcd] | 34.26 ± 2.42 f [hijklm] | -13.69 ± 6.09 f [m] | 3.36 ± 0.28 cd [defghij] | -4.48 ± 7.89 cd [efg] |
| Tomato | Manure biochar+slurry | 7.07 ± 0.13 a [abcd] | 36.95 ± 1.50 ef [fghijklm] | -6.91 ± 3.78 ef [jklm] | 3.63 ± 0.40 bcd [cdefghij] | 3.13 ± 11.34 bcd [defg] |
| Tomato | Manure biochar CO_2_+NH_3_ | 6.83 ± 0.46 abc [bcdefgh] | 51.64 ± 4.60 bc [bcdef] | 30.09 ± 11.58 bc [defghijklm] | 4.55 ± 0.24 ab [abcd] | 29.30 ± 6.71 ab [abcdefg] |
| Tomato | Manure biochar+1x | 7.05 ± 0.03 ab [abcde] | 49.75 ± 3.39 bcd [bcdefg] | 25.32 ± 8.55 bcd [efghijklm] | 4.40 ± 0.48 abc [abcde] | 25.18 ± 13.67 abc [abcdefg] |
| Tomato | Wood biochar | 6.30 ± 0.14 cd [hij] | 35.60 ± 3.47 ef [ghijklm] | -10.33 ± 8.74 ef [klm] | 3.68 ± 0.21 bcd [cdefghij] | 4.62 ± 5.89 bcd [cdefg] |
| Tomato | Wood biochar+slurry | 6.47 ± 0.12 bcd [fghij] | 40.16 ± 2.27 def [efghijklm] | 1.16 ± 5.73 def [ijklm] | 3.77 ± 0.50 abcd [abcdefghij] | 7.18 ± 14.22 abcd [cdefg] |
| Tomato | Wood biochar NH_3_+CO_2_ | 6.32 ± 0.11 cd [hij] | 51.79 ± 1.76 bc [bcde] | 30.45 ± 4.42 bc [defghijkl] | 4.33 ± 0.31 abc [abcdef] | 23.19 ± 8.73 abc [abcdefg] |
| Tomato | Wood biochar+1x | 6.25 ± 0.05 cd [ij] | 57.20 ± 4.41 bc [abcd] | 44.10 ± 11.11 bc [bcdefghi] | 4.86 ± 0.44 a [abc] | 38.26 ± 12.56 a [abcd] |

Table SI 6 Total aluminum (Al), sodium (Na), macronutrients (Ca, K, Mg, P, S), micronutrients (Micronutrients: B, Cu, Mn, Mo, Zn), and heavy metals (Cd, Cr, Pb) in plant shoots. Letters indicate significant differences between amendments within plant and biomass type (*p* < 0.05; n = 4).

| Plant | Amendment | Al | Ca | K | Mg | Na | P | S | Micro-nutrients | Heavy metals |
| --- | --- | --- | --- | --- | --- | --- | --- | --- | --- | --- |
| Shoot |  | (g kg^-1^) | (g kg^-1^) | (g kg^-1^) | (g kg^-1^) | (g kg^-1^) | (g kg^-1^) | (g kg^-1^) | (g kg^-1^) | (mg kg^-1^) |
| Marigold | 0x fert | 4.67 ± 1.25 a | 8.36 ± 0.68 a | 8.07 ± 0.85 a | 5.17 ± 0.47 a | 0.60 ± 0.29 a | 4.44 ± 0.92 a | 3.17 ± 0.63 a | 0.23 ± 0.03 a | 0.45 ± 0.28 a |
| Marigold | 0.25x fert | 4.66 ± 2.07 a | 8.22 ± 0.37 a | 6.84 ± 0.97 a | 5.19 ± 0.45 a | 0.62 ± 0.12 a | 3.85 ± 0.66 a | 2.71 ± 0.55 a | 0.21 ± 0.03 a | 0.28 ± 0.09 a |
| Marigold | 0.5x fert | 4.49 ± 1.17 a | 7.83 ± 0.88 a | 6.37 ± 0.4 a | 5.07 ± 0.83 a | 0.36 ± 0.26 a | 3.28 ± 0.75 a | 2.41 ± 0.45 a | 0.20 ± 0.03 a | 0.40 ± 0.22 a |
| Marigold | 1x fert | 5.47 ± 1.55 a | 8.39 ± 2.08 a | 6.38 ± 0.82 a | 5.64 ± 1.73 a | 0.43 ± 0.34 a | 3.67 ± 0.86 a | 2.53 ± 0.44 a | 0.23 ± 0.04 a | 0.26 ± 0.06 a |
| Marigold | 1.5x fert | 5.61 ± 1.29 a | 7.33 ± 1.12 a | 6.63 ± 0.45 a | 4.80 ± 0.72 a | 0.49 ± 0.26 a | 3.49 ± 0.41 a | 2.62 ± 0.32 a | 0.21 ± 0.03 a | 0.59 ± 0.59 a |
| Marigold | Manure biochar | 4.66 ± 2.53 a | 9.24 ± 1.15 a | 7.64 ± 1.12 a | 5.07 ± 0.58 a | 0.27 ± 0.22 a | 4.40 ± 0.56 a | 3.07 ± 0.24 a | 0.20 ± 0.03 a | 0.26 ± 0.02 a |
| Marigold | Manure biochar+ slurry | 6.63 ± 2.54 a | 8.78 ± 2.03 a | 9.65 ± 2.76 a | 4.95 ± 1.46 a | 0.64 ± 0.38 a | 4.93 ± 1.75 a | 3.50 ± 1.58 a | 0.19 ± 0.04 a | 0.32 ± 0.11 a |
| Marigold | M anure biochar CO_2_+NH_3_ | 2.57 ± 2.3 a | 6.65 ± 4.56 a | 6.96 ± 4.77 a | 3.96 ± 2.80 a | 0.60 ± 0.48 a | 4.06 ± 2.96 a | 2.87 ± 2.06 a | 0.16 ± 0.12 a | 0.20 ± 0.15 a |
| Marigold | Manure biochar+ 1x | 5.65 ± 0.00 a | 7.12 ± 0.00 a | 10.3 ± 0.00 a | 4.13 ± 0.00 a | 0.97 ± 0.00 a | 4.35 ± 0.00 a | 3.16 ± 0.00 a | 0.18 ± 0.00 a | 0.20 ± 0.00 a |
| Marigold | Wood biochar | 3.75 ± 0.75 a | 7.24 ± 0.52 a | 7.56 ± 0.34 a | 3.88 ± 0.21 a | 0.16 ± 0.02 a | 3.19 ± 0.15 a | 2.15 ± 0.08 a | 0.21 ± 0.02 a | 0.17 ± 0.04 a |
| Marigold | Wood biochar+ slurry | 5.10 ± 0.33 a | 6.94 ± 1.11 a | 8.76 ± 1.12 a | 3.95 ± 0.59 a | 0.48 ± 0.24 a | 4.16 ± 0.53 a | 2.67 ± 0.43 a | 0.21 ± 0.03 a | 0.24 ± 0.11 a |
| Marigold | Wood biochar NH_3_+CO_2_ | 6.54 ± 3.73 a | 7.61 ± 0.86 a | 7.96 ± 2.31 a | 4.30 ± 0.44 a | 0.28 ± 0.28 a | 3.44 ± 0.64 a | 2.52 ± 0.68 a | 0.22 ± 0.04 a | 0.42 ± 0.33 a |
| Marigold | Wood biochar+ 1x | 4.69 ± 2.01 a | 7.96 ± 0.84 a | 7.28 ± 0.68 a | 4.68 ± 0.49 a | 0.52 ± 0.24 a | 3.01 ± 0.37 a | 2.23 ± 0.26 a | 0.19 ± 0.05 a | 0.33 ± 0.17 a |
| Radish | 0x fert | 43.04 ± 60.93 a | 23.7 ± 3.85 a | 17.83 ± 4.28 a | 8.78 ± 1.54 a | 7.01 ± 2.19 ab | 2.41 ± 0.30 a | 2.47 ± 0.52 a | 0.44 ± 0.10 a | 0.58 ± 0.12 a |
| Radish | 0.25x fert | 10.62 ± 3.91 a | 22.56 ± 5.53 a | 17.33 ± 5.34 a | 7.52 ± 1.99 a | 7.28 ± 1.70 ab | 2.59 ± 0.77 a | 2.69 ± 0.63 a | 0.41 ± 0.08 a | 0.29 ± 0.12 a |
| Radish | 0.5x fert | 13.12 ± 6.42 a | 22.46 ± 6.00 a | 18.28 ± 4.72 a | 8.62 ± 1.93 a | 9.49 ± 1.20 a | 2.35 ± 0.40 a | 2.58 ± 0.62 a | 0.45 ± 0.04 a | 0.44 ± 0.19 a |
| Radish | 1x fert | 11.49 ± 4.27 a | 24.7 ± 4.50 a | 18.04 ± 4.23 a | 8.77 ± 1.41 a | 9.50 ± 1.88 a | 2.59 ± 0.22 a | 2.29 ± 0.31 a | 0.45 ± 0.03 a | 0.33 ± 0.10 a |
| Radish | 1.5x fert | 20.07 ± 24.15 a | 22.90 ± 5.33 a | 17.61 ± 6.76 a | 7.83 ± 1.57 a | 7.93 ± 2.27 ab | 2.80 ± 1.18 a | 2.96 ± 1.90 a | 0.51 ± 0.17 a | 4.46 ± 7.41 a |
| Radish | Manure biochar | 7.08 ± 1.60 a | 25.53 ± 6.28 a | 20.17 ± 5.05 a | 7.69 ± 1.73 a | 3.72 ± 1.92 b | 2.84 ± 0.68 a | 3.07 ± 1.18 a | 0.46 ± 0.08 a | 0.20 ± 0.05 a |
| Radish | Manure biochar+ slurry | 7.53 ± 2.79 a | 26.7 ± 9.82 a | 19.6x ± 4.77 a | 7.56 ± 2.08 a | 3.77 ± 1.18 b | 2.76 ± 0.34 a | 2.75 ± 0.45 a | 0.45 ± 0.11 a | 0.33 ± 0.12 a |
| Radish | Manure biochar CO_2_+NH_3_ | 10.86 ± 3.06 a | 26.04 ± 4.87 a | 18.46 ± 2.30 a | 8.63 ± 1.57 a | 7.41 ± 4.38 ab | 2.89 ± 0.40 a | 2.58 ± 0.30 a | 0.51 ± 0.08 a | 0.67 ± 0.64 a |
| Radish | Manure biochar+ 1x | 10.47 ± 2.45 a | 26.84 ± 4.56 a | 17.4x ± 3.31 a | 8.67 ± 1.22 a | 6.89 ± 2.04 ab | 2.56 ± 0.47 a | 2.06 ± 0.25 a | 0.48 ± 0.03 a | 0.33 ± 0.12 a |
| Radish | Wood biochar | 8.50 ± 4.75 a | 21.29 ± 6.27 a | 16.3x ± 4.21 a | 6.27 ± 1.86 a | 4.46 ± 2.54 ab | 2.38 ± 0.45 a | 2.46 ± 0.40 a | 0.41 ± 0.08 a | 0.40 ± 0.32 a |
| Radish | Wood biochar+ slurry | 7.89 ± 4.00 a | 18.21 ± 1.38 a | 19.0x ± 3.42 a | 5.90 ± 0.81 a | 5.09 ± 3.02 ab | 2.37 ± 0.57 a | 2.59 ± 0.68 a | 0.40 ± 0.03 a | 0.25 ± 0.16 a |
| Radish | Wood biochar NH_3_+CO_2_ | 20.57 ± 24.53 a | 20.60 ± 2.61 a | 18.17 ± 3.05 a | 6.29 ± 1.23 a | 4.76 ± 0.94 ab | 2.10 ± 0.39 a | 1.90 ± 0.45 a | 0.43 ± 0.04 a | 0.41 ± 0.18 a |
| Radish | Wood biochar+1x | 13.32 ± 6.78 a | 23.71 ± 2.80 a | 20.22 ± 6.06 a | 7.59 ± 0.88 a | 6.40 ± 2.15 ab | 2.40 ± 0.56 a | 2.18 ± 0.49 a | 0.44 ± 0.06 a | 0.59 ± 0.35 a |
| Tomato | 0x fert | 4.53 ± 1.52 a | 12.77 ± 1.27 bc | 12.48 ± 0.49 a | 5.06 ± 0.47 a | 2.48 ± 0.52 ab | 3.39 ± 0.57 abcd | 3.81 ± 0.89 ab | 0.19 ± 0.02 a | 0.10 ± 0.02 a |
| Tomato | 0.25x fert | 4.90 ± 0.37 a | 12.31 ± 0.48 bc | 12.88 ± 0.99 a | 5.08 ± 0.52 a | 2.53 ± 0.33 ab | 3.04 ± 0.22 cd | 3.10 ± 0.35 abc | 0.18 ± 0.01 a | 0.08 ± 0.01 a |
| Tomato | 0.5x fert | 4.78 ± 1.94 a | 10.65 ± 0.87 c | 12.51 ± 1.39 a | 4.42 ± 0.20 a | 2.51 ± 0.49 ab | 2.87 ± 0.28 cd | 3.02 ± 0.47 abc | 0.17 ± 0.02 a | 0.08 ± 0.02 a |
| Tomato | 1x fert | 5.00 ± 0.70 a | 10.2 ± 0.86 c | 12.16 ± 0.08 a | 4.54 ± 0.15 a | 3.08 ± 0.73 ab | 3.11 ± 0.55 cd | 3.58 ± 0.59 abc | 0.16 ± 0.01 a | 0.11 ± 0.04 a |
| Tomato | 1.5x fert | 4.90 ± 2.31 a | 10.15 ± 1.16 c | 12.75 ± 0.49 a | 4.69 ± 0.50 a | 4.33 ± 1.10 a | 3.76 ± 0.71 abc | 3.91 ± 0.92 ab | 0.18 ± 0.02 a | 0.16 ± 0.05 a |
| Tomato | Manure biochar | 6.53 ± 1.42 a | 16.81 ± 1.77 a | 13.29 ± 0.55 a | 5.08 ± 0.50 a | 2.44 ± 0.88 b | 4.36 ± 0.33 a | 4.12 ± 0.52 a | 0.22 ± 0.04 a | 0.14 ± 0.03 a |
| Tomato | Manure biochar+ slurry | 5.90 ± 2.33 a | 14.90 ± 1.60 ab | 13.93 ± 1.23 a | 4.51 ± 0.37 a | 3.41 ± 1.90 ab | 4.22 ± 0.36 ab | 3.95 ± 0.51 a | 0.41 ± 0.46 a | 1.99 ± 3.79 a |
| Tomato | Manure biochar CO_2_+NH_3_ | 5.15 ± 1.33 a | 12.20 ± 0.60 bc | 13.63 ± 0.74 a | 4.27 ± 0.23 a | 2.67 ± 0.31 ab | 3.75 ± 0.51 abc | 3.24 ± 0.33 abc | 0.16 ± 0.01 a | 0.09 ± 0.01 a |
| Tomato | Manure biochar+1x | 4.67 ± 1.33 a | 12.82 ± 1.60 bc | 12.91 ± 0.97 a | 4.23 ± 0.68 a | 1.95 ± 0.50 b | 3.57 ± 0.47 abcd | 3.18 ± 0.58 abc | 0.15 ± 0.02 a | 0.11 ± 0.02 a |
| Tomato | Wood biochar | 5.85 ± 1.67 a | 12.1 ± 1.04 bc | 13.8x ± 0.55 a | 4.22 ± 0.10 a | 2.14 ± 0.22 b | 3.17 ± 0.06 bcd | 3.04 ± 0.38 abc | 0.22 ± 0.01 a | 0.08 ± 0.00 a |
| Tomato | Wood biochar+ slurry | 5.47 ± 2.59 a | 12.03 ± 2.14 bc | 14.04 ± 0.61 a | 4.32 ± 0.60 a | 2.84 ± 0.26 ab | 3.69 ± 0.32 abc | 3.56 ± 0.55 abc | 0.22 ± 0.03 a | 0.23 ± 0.32 a |
| Tomato | Wood biochar NH_3_+CO_2_ | 5.88 ± 2.44 a | 10.6 ± 0.58 c | 12.12 ± 1.42 a | 4.21 ± 0.50 a | 2.29 ± 0.44 b | 2.82 ± 0.31 cd | 2.60 ± 0.06 bc | 0.19 ± 0.01 a | 0.11 ± 0.06 a |
| Tomato | Wood biochar+1x | 5.45 ± 0.59 a | 11.27 ± 1.34 c | 12.59 ± 0.44 a | 4.24 ± 0.50 a | 1.92 ± 0.23 b | 2.59 ± 0.40 d | 2.36 ± 0.24 c | 0.19 ± 0.02 a | 0.08 ± 0.01 a |

Table SI 7 Total aluminum (Al), sodium (Na), macronutrients (Ca, K, Mg, P, S), micronutrients (Micronutrients: B, Cu, Mn, Mo, Zn), and heavy metals (H. metal; Cd, Cr, Pb) in plant roots. Letters indicate significant differences between amendments within plant and biomass type (p < 0.05; n = 4).

| Plant | Amendment | Al | Ca | K | Mg | Na | P | S | Micro-nutrients | Heavy metal |
| --- | --- | --- | --- | --- | --- | --- | --- | --- | --- | --- |
| Root |  | (g kg^-1^) | (g kg^-1^) | (g kg^-1^) | (g kg^-1^) | (g kg^-1^) | (g kg^-1^) | (g kg^-1^) | (g kg^-1^) | (mg kg^-1^) |
| Marigold | 0x fert | 101.07 ± 125.37 a | 4.21 ± 0.43 a | 13.91 ± 3.30 abcd | 2.97 ± 0.63 ab | 22.72 ± 6.44 a | 1.85 ± 0.35 ab | 3.01 ± 0.87 ab | 0.39 ± 0.16 a | 1.66 ± 0.56 a |
| Marigold | 0.25x fert | 59.37 ± 52.51 a | 3.75 ± 0.20 a | 11.08 ± 3.43 abcd | 3.05 ± 1.22 ab | 18.89 ± 3.40 a | 1.43 ± 0.31 abcde | 2.31 ± 0.28 b | 0.30 ± 0.07 a | 1.27 ± 0.33 a |
| Marigold | 0.5x fert | 34.93 ± 16.46 a | 4.01 ± 0.38 a | 9.04 ±  1.83 cd | 3.14 ± 0.57 ab | 17.87 ± 1.77 a | 1.18 ± 0.20 de | 2.04 ± 0.35 b | 0.25 ± 0.05 a | 1.03 ± 0.16 a |
| Marigold | 1x fert | 71.64 ± 75.75 a | 3.97 ± 0.78 a | 8.19 ±  2.82 d | 3.41 ± 1.25 a | 19.93 ± 8.44 a | 1.27 ± 0.30 bcde | 2.10 ± 0.46 b | 0.32 ± 0.16 a | 1.53 ± 1.10 a |
| Marigold | 1.5x fert | 33.85 ± 14.99 a | 3.83 ± 0.22 a | 9.40 ±  1.02 bcd | 2.81 ± 0.47 ab | 19.23 ± 3.41 a | 1.30 ± 0.15 abcde | 2.16 ± 0.14 b | 0.27 ± 0.06 a | 1.11 ± 0.24 a |
| Marigold | Manure biochar | 31.23 ± 14.91 a | 3.96 ± 0.22 a | 17.21 ± 2.44 a | 2.28 ± 0.44 ab | 19.22 ± 4.64 a | 1.76 ± 0.37 abcd | 3.25 ± 0.64 ab | 0.20 ± 0.03 a | 0.59 ± 0.48 a |
| Marigold | Manure biochar+ slurry | 26.78 ± 13.29 a | 3.45 ± 0.38 a | 15.36 ± 3.01 ab | 1.71 ± 0.44 ab | 14.80 ± 3.04 a | 1.48 ± 0.15 abcde | 2.31 ± 0.44 b | 0.19 ± 0.07 a | 0.57 ± 0.41 a |
| Marigold | Manure biochar CO_2_+NH_3_ | 38.5 ± 13.8 a | 3.98 ± 0.44 a | 15.62 ± 2.44 abc | 2.44 ± 0.16 ab | 18.54 ± 1.98 a | 1.93 ± 0.27 a | 3.25 ± 0.65 ab | 0.24 ± 0.07 a | 0.69 ± 0.53 a |
| Marigold | Manure biochar+1x | 28.3 ± 0.00 a | 4.81 ± 0.00 a | 18.22 ± 0.04 a | 2.86 ± 0.00 ab | 26.09 ± 0.02 a | 1.94 ± 0.00 abc | 3.95 ± 0.00 a | 0.28 ± 0.00 a | 0.07 ± 0.00 a |
| Marigold | Wood biochar | 19.17 ± 3.12 a | 3.51 ± 0.70 a | 15.08 ± 2.36 abc | 1.72 ± 0.44 ab | 14.90 ± 4.22 a | 1.44 ± 0.16 abcde | 2.26 ± 0.48 b | 0.34 ± 0.31 a | 0.55 ± 0.20 a |
| Marigold | Wood biochar+ slurry | 31.81 ± 15.54 a | 3.63 ± 0.23 a | 16.40 ± 2.47 a | 1.45 ± 0.47 b | 14.03 ± 2.14 a | 1.75 ± 0.06 abcd | 2.68 ± 0.43 ab | 0.21 ± 0.04 a | 0.54 ± 0.40 a |
| Marigold | Wood biochar NH_3_+CO_2_ | 53.47 ± 44.32 a | 3.65 ± 0.57 a | 13.82 ± 3.03 abcd | 2.22 ± 0.94 ab | 17.44 ± 4.90 a | 1.22 ± 0.24 cde | 2.49 ± 0.64 ab | 0.26 ± 0.11 a | 0.83 ± 0.81 a |
| Marigold | Wood biochar+1x | 34.17 ± 13.82 a | 4.10 ± 0.56 a | 11.22 ± 1.37 abcd | 2.85 ± 0.54 ab | 18.38 ± 2.51 a | 1.12 ± 0.15 e | 2.04 ± 0.42 b | 0.24 ± 0.04 a | 0.65 ± 0.43 a |
| Radish | 0x fert | 26.67± 8.19 a | 4.95 ± 0.79 a | 8.94 ±  2.20 bcd | 4.16 ± 0.43 abcd | 17.17 ± 1.80 a | 2.53 ± 0.29 abcde | 1.46 ± 0.07 ab | 0.20 ± 0.02 a | 0.00 ± 0.00 a |
| Radish | 0.25x fert | 36.78 ± 4.11 a | 4.47 ± 0.29 a | 6.56 ±  1.44 cd | 3.75 ± 0.33 bcde | 14.94 ± 1.55 a | 1.96 ± 0.19 de | 1.41 ± 0.16 ab | 0.23 ± 0.01 a | 0.00 ± 0.00 a |
| Radish | 0.5x fert | 38.10 ± 15.13 a | 4.64 ± 0.46 a | 7.85 ±  2.10 bcd | 3.68 ± 0.32 bcde | 14.89 ± 2.74 a | 1.90 ± 0.41 de | 1.46 ± 0.29 ab | 0.23 ± 0.03 a | 0.00 ± 0.00 a |
| Radish | 1x fert | 50.18 ± 8.43 a | 4.51 ± 0.57 a | 6.05 ±  0.90 d | 3.62 ± 0.56 bcde | 16.23 ± 1.28 a | 1.75 ± 0.46 de | 1.75 ± 0.26 ab | 0.24 ± 0.03 a | 0.00 ± 0.00 a |
| Radish | 1.5x fert | 47.67 ± 29.21 a | 4.43 ± 0.51 a | 9.64 ±  3.56 bcd | 3.70 ± 0.78 bcde | 17.81 ± 3.88 a | 2.46 ± 0.42 bcde | 2.08 ± 0.67 a | 0.25 ± 0.08 a | 0.00 ± 0.00 a |
| Radish | Manure biochar | 32.83 ± 6.62 a | 5.61 ± 0.82 a | 13.82 ± 1.01 abc | 4.86 ± 0.90 ab | 14.21 ± 1.80 a | 3.59 ± 0.36 a | 1.47 ± 0.09 ab | 0.20 ± 0.02 a | 0.00 ± 0.00 a |
| Radish | Manure biochar+ slurry | 30.43 ± 6.97 a | 5.12 ± 0.23 a | 15.01 ± 4.60 ab | 4.62 ± 0.62 abc | 15.72 ± 2.99 a | 3.46 ± 0.45 ab | 1.69 ± 0.35 ab | 0.18 ± 0.01 a | 0.00 ± 0.00 a |
| Radish | Manure biochar CO_2_+NH_3_ | 37.20 ± 11.12 a | 4.98 ± 0.46 a | 9.01 ±  1.71 bcd | 3.95 ± 0.76 abcde | 16.04 ± 3.78 a | 2.35 ± 0.42 cde | 1.30 ± 0.15 b | 0.20 ± 0.01 a | 0.00 ± 0.00 a |
| Radish | Manure biochar+1x | 39.56 ± 19.03 a | 5.75 ± 1.02 a | 10.47 ± 5.72 abcd | 5.24 ± 0.81 a | 17.52 ± 2.19 a | 3.32 ± 0.91 abc | 1.74 ± 0.48 ab | 0.21 ± 0.05 a | 0.00 ± 0.00 a |
| Radish | Wood biochar | 46.10 ± 22.42 a | 5.24 ± 1.05 a | 13.16 ± 3.39 abcd | 3.19 ± 0.51 cde | 13.17 ± 2.41 a | 2.15 ± 0.39 de | 1.33 ± 0.26 b | 0.27 ± 0.09 a | 1.36 ± 2.73 a |
| Radish | Wood biochar+ slurry | 27.82 ± 14.99 a | 4.64 ± 0.63 a | 17.56 ± 3.60 a | 3.03 ± 0.55 de | 13.36 ± 3.82 a | 2.84 ± 0.43 abcd | 1.51 ± 0.23 ab | 0.19 ± 0.04 a | 0.00 ± 0.00 a |
| Radish | Wood biochar NH_3_+CO_2_ | 37.32 ± 8.80 a | 4.59 ± 0.82 a | 8.22 ±  0.48 bcd | 2.91 ± 0.49 de | 16.21 ± 4.47 a | 1.74 ± 0.10 e | 1.25 ± 0.19 b | 0.22 ± 0.03 a | 0.00 ± 0.00 a |
| Radish | Wood biochar+1x | 29.10 ± 9.31 a | 4.69 ± 0.45 a | 7.88 ±  2.52 bcd | 2.49 ± 0.19 e | 12.91 ± 3.02 a | 1.60 ± 0.05 e | 1.15 ± 0.17 b | 0.20 ± 0.04 a | 0.00 ± 0.00 a |
| Tomato | 0x fert | 26.74 ± 8.17 a | 4.95 ± 0.79 a | 8.88 ±  2.24 bcd | 4.16 ± 0.43 abcd | 17.21 ± 1.78 a | 2.53 ± 0.29 abcde | 1.46 ± 0.07 ab | 0.20 ± 0.02 a | 0.00 ± 0.00 a |
| Tomato | 0.25x fert | 36.78 ± 4.11 a | 4.47 ± 0.29 a | 6.60 ±  1.43 cd | 3.75 ± 0.33 bcde | 14.87 ± 1.59 a | 1.96 ± 0.19 de | 1.41 ± 0.16 ab | 0.23 ± 0.01 a | 0.00 ± 0.00 a |
| Tomato | 0.5x fert | 38.07 ± 15.10 a | 4.64 ± 0.46 a | 7.92 ±  2.10 bcd | 3.68 ± 0.32 bcde | 14.91 ± 2.67 a | 1.90 ± 0.41 de | 1.46 ± 0.29 ab | 0.23 ± 0.03 a | 0.00 ± 0.00 a |
| Tomato | 1x fert | 50.24 ± 8.39 a | 4.51 ± 0.57 a | 6.08 ±  0.91 d | 3.62 ± 0.56 bcde | 16.17 ± 1.29 a | 1.75 ± 0.46 e | 1.75 ± 0.26 ab | 0.24 ± 0.03 a | 0.00 ± 0.00 a |
| Tomato | 1.5x fert | 47.70 ± 29.22 a | 4.43 ± 0.51 a | 9.58 ±  3.64 bcd | 3.70 ± 0.78 bcde | 17.80 ± 3.88 a | 2.46 ± 0.42 bcde | 2.08 ± 0.67 a | 0.25 ± 0.08 a | 0.00 ± 0.02 a |
| Tomato | Manure biochar | 32.76 ± 6.61 a | 5.61 ± 0.82 a | 13.84 ±  1.07 abc | 4.86 ± 0.90 ab | 14.22 ± 1.76 a | 3.59 ± 0.36 a | 1.47 ± 0.09 ab | 0.21 ± 0.02 a | 0.00 ± 0.00 a |
| Tomato | Manure biochar+ slurry | 30.42 ± 7.00 a | 5.12 ± 0.23 a | 145.35 ±  4.62 ab | 4.62 ± 0.62 abc | 15.70 ± 3.04 a | 3.46 ± 0.45 ab | 1.69 ± 0.35 ab | 0.18 ± 0.01 a | 0.00 ± 0.00 a |
| Tomato | Manure biochar CO_2_+NH_3_ | 37.19 ± 11.14 a | 4.98 ± 0.46 a | 8.97 ±  1.74 bcd | 3.95 ± 0.76 abcde | 16.01 ± 3.77 a | 2.35 ± 0.42 cde | 1.30 ± 0.15 b | 0.20 ± 0.01 a | 0.00 ± 0.00 a |
| Tomato | Manure biochar+1x | 39.62 ± 18.89 a | 5.75 ± 1.02 a | 10.54 ± 5.73 abcd | 5.24 ± 0.81 a | 17.52 ± 2.23 a | 3.32 ± 0.91 abc | 1.74 ± 0.48 ab | 0.21 ± 0.05 a | 0.00 ± 0.00 a |
| Tomato | Wood biochar | 46.10 ± 22.41 a | 5.24 ± 1.05 a | 13.18 ± 3.39 abcd | 3.19 ± 0.51 cde | 13.22 ± 2.35 a | 2.15 ± 0.39 de | 1.33 ± 0.26 b | 0.27 ± 0.09 a | 1.36 ± 2.73 a |
| Tomato | Wood biochar+ slurry | 27.77 ± 15.02 a | 4.64 ± 0.63 a | 17.61 ± 3.62 a | 3.03 ± 0.55 de | 13.43 ± 3.76 a | 2.84 ± 0.43 abcd | 1.51 ± 0.23 ab | 0.19 ± 0.04 a | 0.00 ± 0.00 a |
| Tomato | Wood biochar NH_3_+CO_2_ | 37.30 ± 8.81 a | 4.59 ± 0.82 a | 8.19 ±  0.53 bcd | 2.91 ± 0.49 de | 16.24 ± 4.48 a | 1.74 ± 0.10 e | 1.25 ± 0.19 b | 0.22 ± 0.03 a | 0.00 ± 0.00 a |
| Tomato | Wood biochar+1x | 29.12 ± 9.31 a | 4.69 ± 0.45 a | 7.88 ±  2.47 bcd | 2.49 ± 0.19 e | 12.89 ± 2.99 a | 1.60 ± 0.05 e | 1.15 ± 0.17 b | 0.20 ± 0.04 a | 0.00 ± 0.00 a |
